# Salt flat microbial diversity and dynamics across salinity gradient

**DOI:** 10.1038/s41598-022-15347-8

**Published:** 2022-07-04

**Authors:** Khaled M. Hazzouri, Naganeeswaran Sudalaimuthuasari, Esam Eldin Saeed, Biduth Kundu, Raja Saeed Al-Maskari, David Nelson, Alya Ali AlShehhi, Maryam Abdulla Aldhuhoori, Dhabiah Saleh Almutawa, Fatema Rashed Alshehhi, Jithin Balan, Sunil Mundra, Mohammad Alam, Kourosh Salehi-Ashtiani, Michael Purugganan, Khaled M. A. Amiri

**Affiliations:** 1 Khalifa Center for Genetic Engineering and Biotechnology, United Arab Emirates University, P.O. Box. 15551, Al Ain, UAE; 2 Department of Biology, College of Science, United Arab Emirates University, P.O. Box. 15551, Al Ain, UAE; 3 Center for Genomics and Systems Biology, New York University, P.O. Box. 129188, Abu Dhabi, UAE; 4 Center for Genomics and Systems Biology, New York University, New York, NY 10003 USA

**Keywords:** Genetics, Ecology

## Abstract

Sabkhas are hypersaline, mineral-rich, supratidal mudflats that harbor microbes that are adapted to high salt concentration. Sabkha microbial diversity is generally studied for their community composition, but less is known about their genetic structure and heterogeneity. In this study, we analyzed a coastal sabkha for its microbial composition using 16S rDNA and whole metagenome, as well as for its population genetic structure. Our 16S rDNA analysis show high alpha diversity in both inner and edge sabkha than outer sabkha. Beta diversity result showed similar kind of microbial composition between inner and edge sabkha, while outer sabkha samples show different microbial composition. At phylum level, Bacteroidetes (~ 22 to 34%), Euryarchaeota (~ 18 to ~ 30%), unclassified bacteria (~ 24 to ~ 35%), Actinobacteria (~ 0.01 to ~ 11%) and Cyanobacteria (less than 1%) are predominantly found in both inside and edge sabkha regions, whereas Proteobacteria (~ 92 to ~ 97%) and Parcubacteria (~ 1 to ~ 2%) are predominately found in outer sabkha. Our 225 metagenomes assembly from this study showed similar bacterial community profile as observed in 16S rDNA-based analysis. From the assembled genomes, we found important genes that are involved in biogeochemical cycles and secondary metabolite biosynthesis. We observed a dynamic, thriving ecosystem that engages in metabolic activity that shapes biogeochemical structure via carbon fixation, nitrogen, and sulfur cycling. Our results show varying degrees of horizontal gene transfers (HGT) and homologous recombination, which correlates with the observed high diversity for these populations. Moreover, our pairwise population differentiation (Fst) for the abundance of species across the salinity gradient of sabkhas identified genes with strong allelic differentiation, lower diversity and elevated nonsynonymous to synonymous ratio of variants, which suggest selective sweeps for those gene variants. We conclude that the process of HGT, combined with recombination and gene specific selection, constitute the driver of genetic variation in bacterial population along a salinity gradient in the unique sabkha ecosystem.

## Introduction

Sabkhas (Arabic) are salt flats that dominate the terrestrial landscape in arid tropical and subtropical regions^1^. They are distributed across North Africa, the Gulf States, Mexico, the USA, and Australia, and the United Arab Emirates (UAE) contains the highest percentage of sabkha area per country^2^. Sabkhas are of two types: coastal and inland sabkhas (i.e., continental sabkha). Coastal sabkhas are generally found along coastline areas of arid regions, formed by seawater evaporation and contain siliciclastics or carbonate sediments, whereas inland sabkhas are usually flanked by towering dunes^3^. Coastal sabkha in the Gulf coast of the Arabian peninsula are Pleistocene dunes that has developed over the past 7000 years through wind erosion of pre-existing dunes and progradation of inter- and supratidal carbon sediments^4^.

Sabkha soil is near neutral in pH and has soluble and non-soluble salt deposits^5,6^, that includes high concentrations of Na, Mg, Ca, K, SO_4_, HCO_3_, and especially Cl^−^^7^; A salinity gradient is marked from the internal areas of sabkhas to their outer edges. The high concentrations of Cl^−^ are remnants of past hydrological activity and mirror Cl^−^ deposits found in the analogous salt flat sites of Mars^8–10^. In addition, ionic composition of soils, mineralogical, and structural, sabkhas are considered excellent models for where petroleum is formed^11,12^.

Despite the extreme environmental conditions within sabkhas, they provide a habitat for various microbial life forms^13^. Microbial communities interactions of coastal sabkhas are expected to play biogeochemical roles in the maintenance of this ecosystem^14^. Earlier studies using 16S, 18S rDNA sequencing to profile microorganisms in sabkhas (culturable and unculturable) have been conducted in Saudi Arabia and other Middle Eastern countries^1,5,6,13,15,16^. Although, these studies are useful at elucidating microbial communities and their ecological role, they were not sufficient to examine genetic variation within sabkha microbial populations^17^. Since most of these identified sabkha microorganisms from earlier studies lack genomic information, there is a need for whole genome metagenomic analysis to further understand the genomic structure of the sabkha ecosystem^18–21^. Sequence variation within assembled metagenomes will help elucidate the evolutionary forces operating in the natural populations of sabkhas.

In this study, we examined the abundance of microbial communities in coastal sabkha and sabkha-shore regions in Abu Dhabi, UAE using 16S rDNA, and whole-genome metagenome analysis to elucidate the genetic heterogeneity of microbial species. Based on the 16S rDNA based prokaryotic microbial profile, we identified unusual coastal sabkha-specific microbial communities, and it is consistent with the whole genome metagenome analysis. Out of 225 assembled microbial metagenomes, we analyzed 82 of the most abundant assembled genomes at the order and class taxonomic levels. We observed higher diversity for some microbial populations on the inner regions of the sabkha as well as the outside it. However, the overall population diversity was higher within the sabkha. Our results show genetic structure over local spatial scales, different level of homologous recombination for different species as well as gene-specific selective sweeps. The results enhance the understanding of the ecological roles, salt stress tolerance mechanisms, and potential applications of sabkha microbial genes.

## Methods

### Sabkha soil sample collection, DNA isolation and sequencing

**Figure 1:**
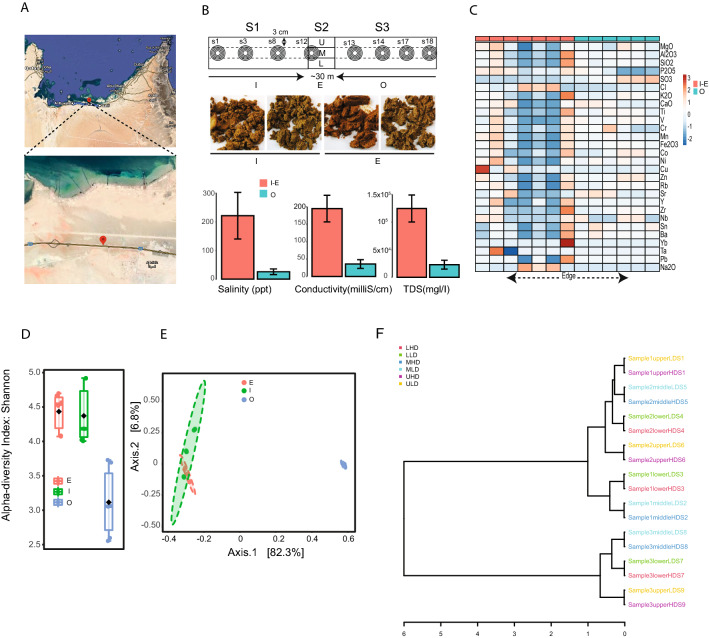
Sampling design, mineral, and 16S diversity analysis. (**A**) Sampling sites for coastal sabkha at Al Mirfa Abu Dhabi (UAE) (top panel). A zoom on the sampling site is shown (below panel), where you see the color difference of salt to sand of coastal sabkha. (**B**) Schematic drawing of the intersection of salt and sand in a coastal sabkha (top panel). Sampled 16S regions are S1 (inner), S2 (edge) and S3 (outer) sabkha. The sampling was done in duplicates at a 3 cm depth, where the core sample was divided, as high (upper layer), middle (middle layer) and bottom (lower layer). For whole metagenome, sampling was done in a 0.5 m interval from the edge (3 m length), starting with s12, then s8, s3, s1 toward the inner sabkha defined as (I–E). Similarly, this was done toward the outside with 0.5 m interval with s13, s14, s17, and s18 and defined as (O). A picture of different samples collected, which shows the copper enriched samples on the inside to edge (middle panel). The lower panel depicts the difference in salinity between inside and outside. (**C**) Heatmap of the different minerals in sabkha from inside to outside. Color band represent logarithm values. (**D**) Shannon diversity index for inner and outer sabkha. (**E**) Bray–Curtis beta diversity presented as a principal component for inner and outer sabkha. (**F**) Dendrogram showing the clustering of samples (Sample 1: inner; Sample 2: edge and Sample 3: Outer).

In this study, soil samples were collected from the Abu Dhabi sabkha (24°04′01.1″N 53°22′28.5″E, Al Mirfa region), UAE, in February 2018 (18/02/2018) (Fig. 1A). We sampled three sites—one in the inner sabkha area (I site; S1), one at the edge of the sabkha (E site; S2) and one outside sabkha (O site; S3). All samples consists of duplicates. Each sample consisted of vertical three layers (upper, middle, and lower layer) as indicated in Fig. 1B. In total, 18 samples were collected from the sabkha for DNA isolation (16S rDNA analysis) as well as soil analysis.

From the collected soil samples, DNA isolation was carried out using Qiagen (Hilden, DE) Power Soil kit as per the manufacturer’s instruction. Isolated DNA quality was confirmed using agarose gel electrophoresis, and DNA concentration was measured with NanoDrop spectrophotometer (ThermoFisher Scientific, Waltham, MA) and Qubit 2.0 fluorometer (ThermoFisher Scientific). In addition, DNA samples were amplified using universal V3–V4 primers (16S rDNA primer 341F: CCTACGGGNGGCWGCAG and 805R: GACTACHVGGGTATCTAATCC), and sequencing libraries were prepared for the PCR amplicons and sequenced using Illumina MiSeq platform (300 bp PE chemistry).

### 16S rDNA-based (V3–V4 region) prokaryotic microbial profiling

Quality of the 16S rDNA raw Illumina data was confirmed using the FastQC tool^22^. The sequencing adapter and low quality regions found in the reads were removed using Scythe v0.9^23^ and Sickle v1.33^24^ programs. Trimmed reads were further analyzed using DADA2 metagenomic analysis pipeline^25^. During DADA2 analysis, filterAndTrim (truncLen = c (250, 230), maxN = 0, maxEE = c (2,2), truncQ = 2) function was used for the PE reads length trimming. During trimming, forward and reverse reads were trimmed to 250 and 230 bps, respectively. Reads with lengths less than 250 bps (forward reads) and length less than 230 bps (reverse reads) were removed from the analysis, and the mergePairs function was used to merge forward and reverse reads. Function assignTaxonomy was used to map the merged reads against the 16S RNA database (silva_nr_v138)^26^, and biom file was created. The biom file with metadata file was used in a web-based tool MicrobiomeAnalyst^27^. Alpha diversity was generated with the Chao1, and Shannon diversity index and beta diversities was generated using the Bray–Curtis dissimilarity distance distribution. ANOVA test for alpha diversity across sampled layers was used to test for significance between inside and outside the sabkha, while PERMANOVA test was used for beta diversity analysis. Taxonomic identity was generated as a heatmap of abundance at the phylum and order level inside and at the edge of the sabkha, and outside, as well as at different depths between the upper (upper and lower), middle (upper and lower) and lower (upper and lower).

### Sabkha soil property and mineral analysis

One hundred grams of sabkha soil from the collected sample was dissolved by adding 200 ml of distilled water and stirred for an hour, filtered and salinity, pH, TDS and conductivity was measured using a multiparameter Hack instrument (USA). For mineral analysis, sabkha soil samples were dried, and the minerals present in the samples were measured using XRD PANalytical and XRF Epsilon instruments.

### Metagenome DNA isolation, sequencing and analysis

For whole genome metagenome analysis, 8 different sabkha sites were sampled (s1, s3, s8, s12 for inner) and (s13, s14, s17, s18 for outer) (Fig. 1B). Each site is a pool of 3 replicate samples. From the collected soil samples, DNA isolation was carried out using Qiagen (Hilden, DE) power soil kit as per manufacturer’s instruction. Eight shotgun whole genome metagenome libraries were generated using NEBNext^®^ UltraTM DNA Library Prep Kit (New England Bioloabs, Ipswich, Massachusetts, United States) according to the prescribed protocol. Each of the 8 sampled sites were spaced equally with half of meter from inside and from outside (Fig. 1B). Furthermore, libraries were sequenced with Illumina NovaSeq 6000 (150-bp paired end reads).

Metagenome read quality and trimming were performed in FastQC and Trimmomatic^28^. Metagenome analysis was carried out using the MetaWRAP v1.3.2 pipeline^29^. Metagenome assembly was performed using Meta-SPAdes v3.13 assembler^30^. The initial read and contig level species diversity was estimated using kraken2 module implemented in MetaWRAP pipeline (-t 90 -s 50,000,000). Furthermore, we used binning, bin_refinement, quant_bins and reassemble bins modules to separate the contigs into separate bacterial contig bins (minimum completeness > 50% and contamination level < 10%). The gene annotation of the assembled genome was performed using Prokka v1.14.5^31^. Biosynthetic gene clusters as well as transporters found in the assembled bacterial genomes were identified using antiSMASH v5.1.1 tool^32^. For downstream population genetic analysis of whole metagenome, bin assemblies were dereplicated using dRep v3.2.2^33^ and clustering threshold of –sa 0.97 was used and then were filtered with ChekM^34^ to have a dereplicated species level of 97% Average Nucleotide Identity (ANI) for read mapping with 74% completeness, less than 10% contamination and a minimum of 1% abundance for each pooled triplicate sample/per site on inner and outer sabkha. The final set of contigs used in the downstream analysis is reliably and independently assembled for a species in multiple samples/sites. We looked at the individual microbiome contribution to community functions, using a replication index iRep v1.1.14^35^. The estimate was performed on the realigned reads to the assembled meta-genomes bins. This method uses an algorithm that assumes a bi-directional replication from a single origin accounting for coverage changes in fragmented genomes. For instance an iRep of 2 means that the population of cells are all actively replicating, whereas an iRep of 1.25 implies that a quarter of them are active^35^. Viral and fungal community profile of the sabkha was identified using MiCoP tool^36^. MetaPhlAn v3.0 pipeline^37^ was used for taxonomic profiling of the meta-genome shot gun reads with and without inclusion of viral community. Pavian^38^ was used to visualize the abundance and diversity at various taxonomic levels.

Genes involved in metabolic pathways and biogeochemical cycle were identified from the assembled metagenome using METABOLIC^39^ pipeline. The characterization of markers was identified using KEGG^40^ and curated custom protein HMM databases with motif confirmation using validated conserved residues in proteins. Diagrams representing metabolic pathways are generated for individual genomes as well as summary of the biogeochemical cycling processes.

### Horizontally transferred gene regions using whole meta-genome assemblies

To detect horizontal gene transfers (HGTs) inside and outside sabkha, we used the HGT MetaChip v1.10.3 pipeline (metagenomics community-level HGT identification pipeline)^41^, which uses a combination of best-match and phylogeny for HGT detection. The set of metagenome assemblies (bins) were used as an input. We used GTDB-Tk tool^42^, for taxonomic classification of our input genomes using phylogenetically calibrated the Genome Taxonomy Database (GTDB)^43^. We defined the grouping of our taxonomic analysis of HGT at the order level. Enrichment analysis of PFAMs of horizontally transferred genes were scanned for enriched biological, cellular and molecular categories using an online tool (https://supfam.mrc-lmb.cam.ac.uk/SUPERFAMILY/cgi-bin/dcenrichment.cgi). Plots of PFAM relationship terms are generated with R using Revigo^44^.

### Diversity analysis

All metagenomic reads per sample/site were mapped using bwa^45^ to indexed databases of dereplicated genomes obtained from inner and outer sabkha. Uniquely mapped reads to the representative genomes were used for analysis. The generated BAM files were filtered for uniqueness in which both reads mapped to the same scaffold with mappability of 96% match and mapping quality of mapq > 1 to their representative reference genomes. To achieve this, we used published custom script filter_reads.py available from (https://github.com/alexcritschristoph/soil_popgen). We have conducted quality check comparison where we compared ANI genes with 96% to a 98% ANI cutoff. SNPs were called for each population for the pooled samples/site at frequencies > 5% using a simple null model that assumes a FDR < ~ 10^–6^.

Population diversity analysis was carried out using reproducible custom scripts that were fitted to our data from https://github.com/alexcritschristoph/soil_popgen. Nucleotide diversity was calculated as the expected frequency difference between two reads at each separate position with minimum 20 × coverage within each sample and averaged across genes. We quantified if there is any bias in changing coverage on nucleotide diversity, where we calculated nucleotide diversity at subsampled coverage for each genomic position. SNPs were assigned as synonymous and nonsynonymous using reproducible scripts from https://github.com/alexcritschristoph/soil_popgen on gene calls annotated with Prodigal v2.6.1^46^.

### Linkage disequilibrium, population dynamics and selection tests

Linkage was calculated for all pairs of segregating sites of mapped reads with minimum spanning of 30 high quality base pair reads. *R*^2^ and *D*′ was calculated using equation from VanLiere^47^. We used mcorr package^48^ on synonymous third position codon sites to calculate the rate of recombination to mutation (δ/μ) for each population. Population structure, Fst, which measures the difference in allele frequencies between two populations, was calculated on segregating sites across inside, outside and across edge of sabkha. This was done using 20 × coverage per sampled site using the method by Hudson^49^, as recommended by Bhatia et al.^50^ using the python package scikit-allel^51^. Genes with coverage exceed two standard deviations were excluded from the analysis. Mean Fst was determined as the ratio of averages for each gene. The average nucleotide diversity of high Fst compared to genomic average of each species was done using *t* tests and similarly the average linkage of high Fst compared to genomic linkage average was calculated using *t* test in R (https://www.r-project.org/).

## Results

### Sabkha soil analysis

Soil biochemical analysis shows higher concentration of salt in the middle (average of 200 ppt) which decreases to 20 ppt outside of the sabkha (Fig. 1B). Soil conductivity and total dissolved solids (TDS) were positively correlated with salinity (r = 0.8, p < 0.05). Cl^−^; Na_2_O was only abundant in the middle, whereas Cu, Fe_2_O_3_, P_2_O_5_, SiO_2_ and Al_2_O_3_ were abundant in the inner and edge of the sabkhas compared to the outside. In contrast, SO_3_ was more pronounced on the outside (Fig. 1C, Supplementary Tables 1, 2).

### Dynamic shift in metagenomic profile across sabkha salinity gradient

Species diversity within the samples (alpha-diversity) were measured by Shannon diversity index method. The highest diversity was identified in the high salt regions Site I (diversity: 4–4.8) and the edge of sabkha (E) samples (diversity: 4.1–4.6) (Fig. 1D). The lowest microbial diversity (2.5–3.8) was observed in the samples collected from the combined sand and overflow sabkha region (outside). Alpha diversity measures for upper, lower and middle across the eighteen samples show no significant difference among layers (ANOVA F-value = 0.86572, p = 0.53) (Supplementary Fig. 1). The difference in the diversity is only observed between inside and outside the sabkha, which correlates with the salt gradient (ANOVA F-value = 18.962, p-value = 7.8 × 10^–5^). Diversity between the samples (Beta diversity) analysis of the sabkha microbial composition along the three different sites (I, E, O) identified similar microbial populations between inside and edge sabkha samples, clustered together in a PCoA analysis. Samples collected from outside sabkha region formed a separate cluster in the PCoA analysis (Fig. 1E,F) (PERMANOVA F-value = 45.062, R^2^ = 0.857, p-value < 0.001).

**Figure 2:**
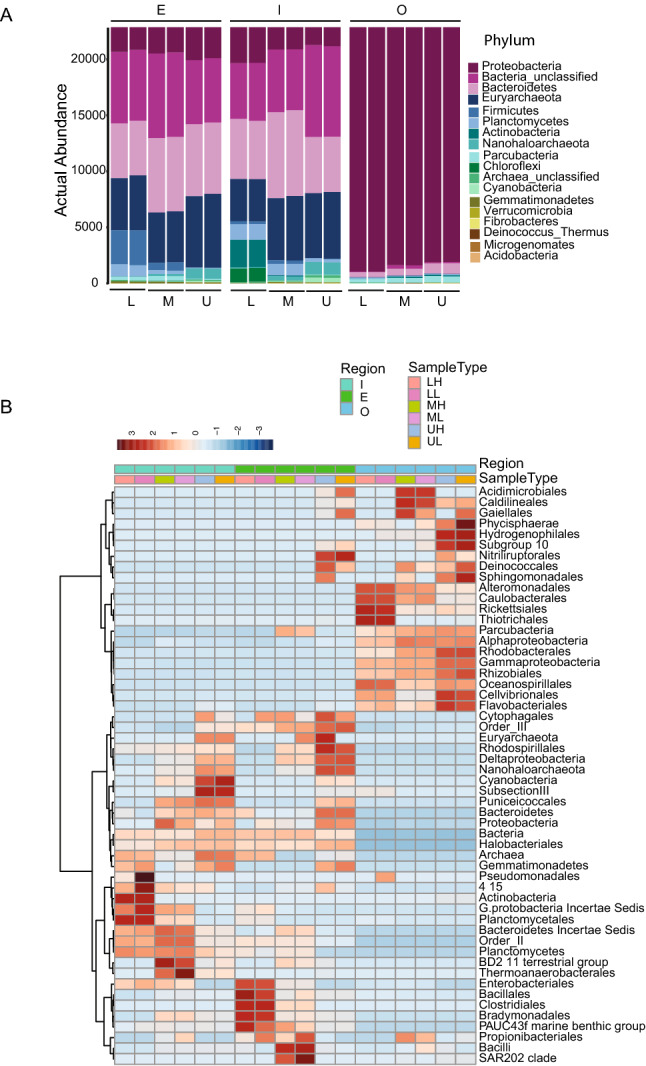
(**A**) Phylum level actual abundance of the inner and outer sabkha as well as different 3 cm layers (L: low; M: medium; U: upper). (**B**) Heatmap clustering of bacterial abundance as the order level, by type as well as by region inner and outer sabkha. Logarithmic value of OTUs abundance is represented by a color bar on top of the heatmap.

Taxonomic identity shows that there is shift in abundance between inside and outside the sabkha (Fig. 2A,B) and it is correlated with the salt gradient (R = 0.85, p < 0.05). At phylum level, Bacteroidetes (~ 22 to 34%), Euryarchaeota (~ 18 to ~ 30%), unclassified bacteria (~ 24 to ~ 35%), Actinobacteria (~ 0.01 to ~ 11%) and Cyanobacteria (less than 1%) are predominantly found in both inside and edge sabkha regions, whereas Proteobacteria (~ 92 to ~ 97%) and Parcubacteria (~ 1 to ~ 2%) are predominant in outer sabkha, Cyanobacterial classes were found to be over-represented inside the sabkha compared to the outside. Several archaeal taxa, as well as some unclassified bacteria, were rich in the inner and edge sabkha samples, while halophilic bacteria were overrepresented on the inside hypersaline region of the sabkha (Fig. 2A). Halobacteriaceae, Actinobacteria, Planctomycetaceae, Bacteroidetes and Planctomycetes are found more abundant in inside sabkha. Gammaproteobacteria, Rhodobacteraceae, Alphaproteobacteria, Rhizobiales and Oceanospirillales are found more abundant in the hyposaline outer sabkha. We see a shift in the Proteobacteria structure (Fig. 2B, Supplementary Fig. 2), where Delta/Epsilon Proteobacteria were more abundant in the hypersaline region inside the sabkha, while Gammaproteobacteria, and Alphaproteobacteria were more abundant in the hyposaline outer sabkha (Fig. 2B). Interestingly, we see similar taxonomic groups in the middle and lower layer of inside sabkha and less groups in the upper salt crust of each section (Fig. 2B), but not on the outside the sabkha.

**Figure 3:**
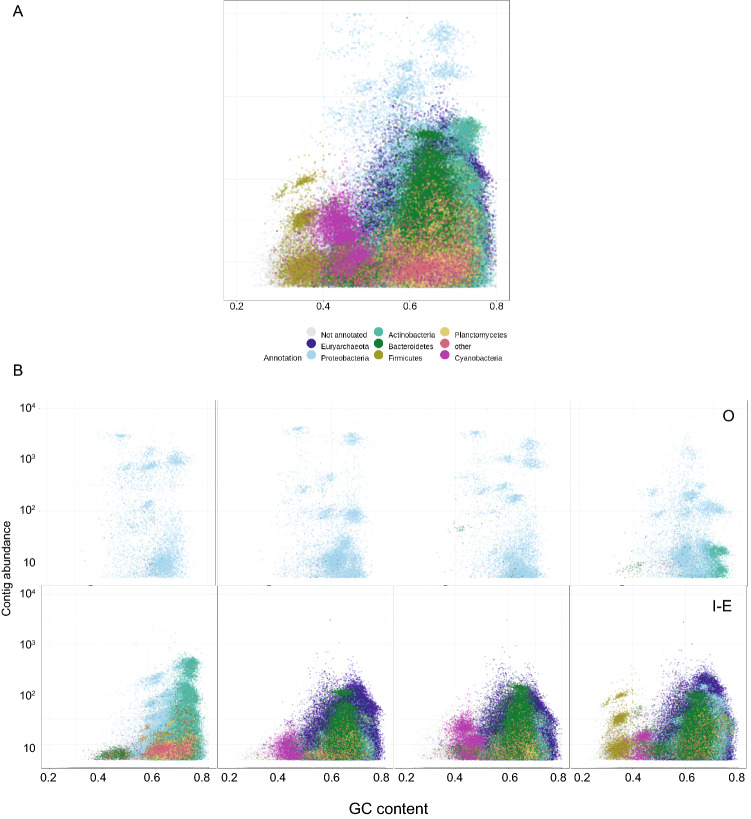
Blob plot of abundance versus GC content. (**A**) Blob plot of inside-edge and outside combined depicting different phylum’s abundance. (**B**) Separate Blob plot of inside-edge and outside sabkha.

**Figure 4:**
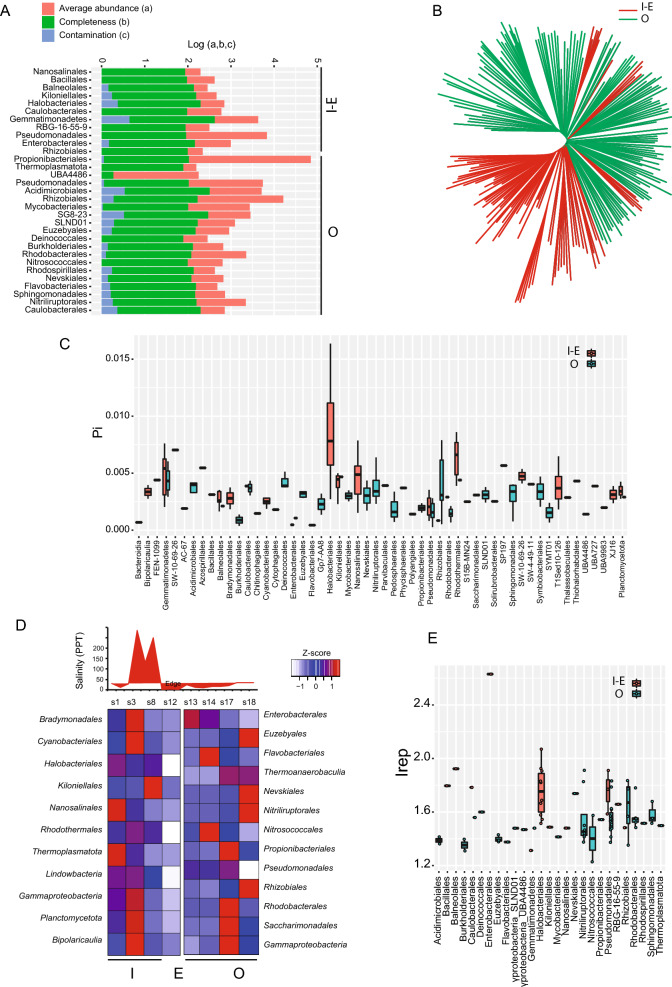
Whole metagenome resolved genome assemblies’ analysis. (**A**) Barplot of the 225 whole metagenome resolved genome assemblies across the different taxonomic order level, plotted by logarithm of completeness, abundance, and contamination. (**B**) kmer-based phylogenomic tree of inside and outside sabkha. (**C**) Nucleotide diversity (Pi) for the different taxonomic order level of inside and outside sabkha. (**D**) Salinity as parts per thousand (PPT) plots across inner and outer sabkha (top panel). Dereplicated genomes for inner and outer sabkha with minimum 1% abundance across sites samples per population, which represented by a Z-score of the mean (bottom panel). (**E**) Replication index (Irep) for the mapped reads to the actual representative genomes, where value above 1 represent the percentage of replicating cell in the population (e.g., 1.2, means 20% of cells are replicating).

For the sabkha whole metagenome assembly, the 4 sites sampled on inside, edge (s1, s3, s8, s12) and the 4 sites outside (s13, s14, s17, s18) (Fig. 1B) were used for assembly to resolve the extent of genome completeness. In total ~ 830 million PE reads (150 bp) were generated (Supplementary Table 3) for the metagenome assembly, resulting in 664,455 contigs (final genome assembly length ~ 2.3 Gb) with the N50 value of 3.945 Kb (Supplementary Table 4). All the assembled contigs were taxonomically classified (at phylum level) based on the sample location. The contigs related to the phyla Euryarchaeota, Bacteroidetes, Actinobacteria, Planctomycetes, Cyanobacteria, and Firmicutes are predominantly found in the inside sabkha samples, whereas phyla Proteobacteria is predominant in outside sabkha region (Fig. 3A,B). In addition, contigs were binned into 225 genomes across the sabkha (Fig. 4A). Assembled genome bacterial size ranges from ~ 0.5 to ~ 9.3 Mb, with the N50 of the assembled genome from ~ 2 Kb to ~ 1 Mb (Supplementary Table 5). From the assembled bins, we annotated CDS, tRNA, rRNA and tmRNA sequences (Supplementary Table 5). In total, 651,373 protein coding (CDS) and 6698 noncoding genes were annotated from the assembled genome. The phylogenetic tree generated using assembled genomes is shown in (Fig. 4B).

Taxonomically resolved genomes show high nucleotide diversity (π) levels on the inside compared to the outside of the sabkha (Fig. 4C). We observed comparable taxonomic microbial profiles using whole metagenome and 16S rDNA analysis. Interestingly, Halobacteria have the highest diversity on the inside followed by Rhodotermales, whereas Rhizobiales and Pseudomonadales are highest from the outside (Fig. 4C).

The pairwise genome-wide ANI (Average Nucleotide Identity) and alignment coverage of 125 dereplicated but quality-filtered genomes across the sabkha showed a decrease of pairwise ANI at ~ 97% (Supplementary Fig. 3), which is comparable to other reported thresholds in bacterial species^52^. The clustering of genomes using dRep into groups of species-like populations using ANI = 97% (Supplementary Fig. 4) was done and genomes with > 74% completeness and less than 10% contamination kept for further analysis. The focus on abundant populations/sites with more than 1% abundance resulted in 129 dereplicated genomes with a least 3 genomes per population summarized in Fig. 4D. Using metagenome-assembled genomes, the measure of Irep index show that the inside and outside is fully replicating (Fig. 4D). There is statistically significant difference (*t* test p = 2.43 × 10^−5^) between Irep for overall taxonomic order group from hypersaline inside (1.744 ± 0.274) compared to hyposaline outside (1.527 ± 0.145) sabkha (Supplementary Table 6). On the inside, we observe Halobacteriale with an average of 1.754 ± 0.184, while on the outside, Pseudomonadales has 1.554 ± 0.124, which is significantly lower (*t* test p = 0.02) than inside sabkha. Rhizobiales on the outside is replicating more than the overall outside with Irep around 1.632 ± 0.218 (Fig. 4E). We found no correlation between Irep and Pi for the different taxonomic order (Pearson R = − 0.12, p = 0.53) (Supplementary Fig. 5, Supplementary Table ,6), which suggest involvement of replication of these bacteria in nutrient cycling of sabkha (Supplementary Fig. 7).

### Nucleotide diversity in the sabkha

**Figure 5:**
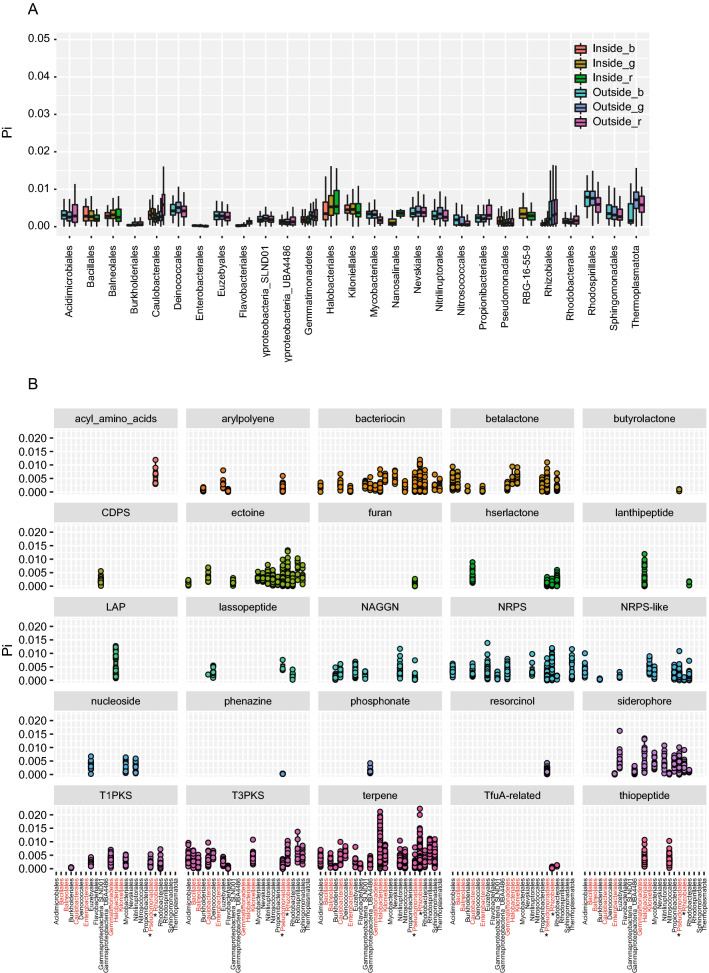
Nucleotide diversity analysis within sabkha populations. (**A**) Nucleotide diversity (Pi) for different taxonomic order level for genic, biosynthetic as well as ribosomal genes, represented by (_b: biosynthetic; _g: genic; _r: ribosomal). (**B**) Nucleotide diversity (Pi) for different class of biosynthetic genes for different taxonomic order level for the different metagenomic bins. Red labels identify the inner sabkha bins and star (*); identify the presence in both inner and outer sabkha.

The overall combined microbial population nucleotide diversity (π) was highly significant for all inner microbial communities compared to outside the sabkha (Fig. 5A) as is evident for each sampling site on the inner (s1, s3, s8, s12) compared to outer (s13, s14, s17, s18) (*t* test Bonferroni p < 0.05) (Supplementary Fig. 8). Clearly, *Halobacteriales* and *Pseudomonadales* populations have high diversity from inside the sabkha, while *Rhizobiales* and *Pseudomonadales* have highest diversity from outside the sabkha (Supplementary Fig. 8). The comparable diversity of combined as well as per site samples suggests that diversity persists within soils at even finer scale. We also found comparable ranges of per gene nucleotide diversity values (Pearson R = 0.9, p < 0.05) (Supplementary Fig. 9) for different populations at 96% and 98% ANI (Supplementary Fig. 10).

We found in almost all sabkha populations that ribosomal genes have lower nucleotide diversities that the average for genes in the genome (Fig. 5A), consistent with purifying selection^53^. An exception is for the inner sabkha *Halobacteriales* population, and outer sabkha populations such as *Propionibacteriales*, *Pseudomonadales*, *Rhizobiales*, and *Rhodobacteriales,* which have higher ribosomal nucleotide diversities, suggesting that they play an adaptive role in diverse ecological niches and microenvironment. Moreover, biosynthetic genes annotated with antiSMASH have significantly higher diversity than the genome average (pairwise *t* test Bonferroni p < 0.05), except for *Halobacteriales, Thermoplasmatota, Pseudomonadales*, and *Rhizobiales.*

When we looked at the diversity of different classes of biosynthetic genes from inside and outside the sabkha, we find unique patterns of biosynthetic gene class counts (Supplementary Fig. 11, Fig. 5B). Overall shared classes of inside and outside the sabkha include terpene, bacteriocin, betalactone, hserlactone, T3PKS, T1PKS, siderophores and ectoine (Supplementary Fig. 11, Fig. 5B). Inside the sabkha, however, we observe for *Halobacteriales* LAP (linear azol (in) e-containing peptides) that are synthetized in ribosomes, lanthipeptide and butyrolactones. On the other hand, we observed in outside of the sabkha, we see biosynthetic genes from different unique populations classes, such arylpolyene, CDPS, furan, lassopeptide, nucleoside, NRPS (NonRibosomal Peptide-Synthetase), phenazine, phosphonate, resorcinol and TfuA-related (Supplementary Fig. 11, Fig. 5B).

### Horizontal gene transfer, homologous recombination and sabkha microbial diversity

**Figure 6:**
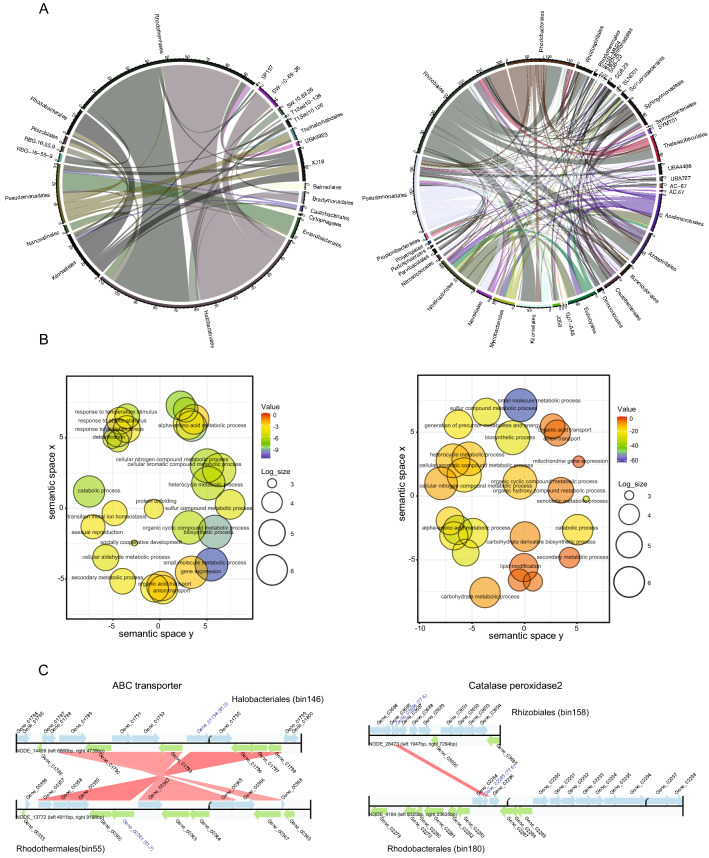
Horizontal gene transfer at the taxonomic order level for inner and outer sabkha. (**A**) MetaChip summary Circos plot of horizontal gene transfer (HGT) at the order level for inner (left panel) and outer (right panel). The thickness of the connecting band represents that more transfer is happening between these groups. (**B**) PFAM enrichment analysis for inner (left panel) and outer (right panel) using dcGO. The results are plotted as a sematic space scatter plot by the logarithmic p value represented by a color bar chart and logarithm size of the PFAM numbers. (**C**) Example of inner (left panel) HGT between *Halobacteriales* and *Rhodothermales* for ABC transporter, and outer (right panel) for a Catalase peroxidase2 between *Rhizobiales* and *Rhodobacteriales*.

Our analysis at the order level show that there is extensive horizontal gene transfer on the inside and outside the sabkha (Fig. 6A). On the inside, there is an exchange of genetic information between different levels especially *Halobacteriales*–*Rhodotermales*, reaching close to 42 HGT events (Fig. 6A, Supplementary Fig. 12). On the other hand, our analysis shows 30–47 HGT events between *Rhizobiales*–*Rhodobacteriales*, *Kiloniellales*–*Rhizobiales* and *Pseudomonadales*–*Nevskiales* outside the sabkha (Fig. 6A, Supplementary Fig. 12).

Using PFAM domains, we observed enriched transfer of abiotic stress genes, detoxification, biosynthetic of small molecules, transport, homeostasis and transport genes associated with HGT events (hypergeometric test, p < 0.05) (Fig. 6B). Two examples of HGT are highlighted in Fig. 6C. Interestingly, we see a gene for social cooperative development inside the sabkha. Cooperation among bacteria is a common strategy of interaction via cell–cell signaling, termed ‘quorum sensing’ (QS), which is an evolutionary stable strategy under extreme abiotic or biotic stress^54^.

**Figure 7:**
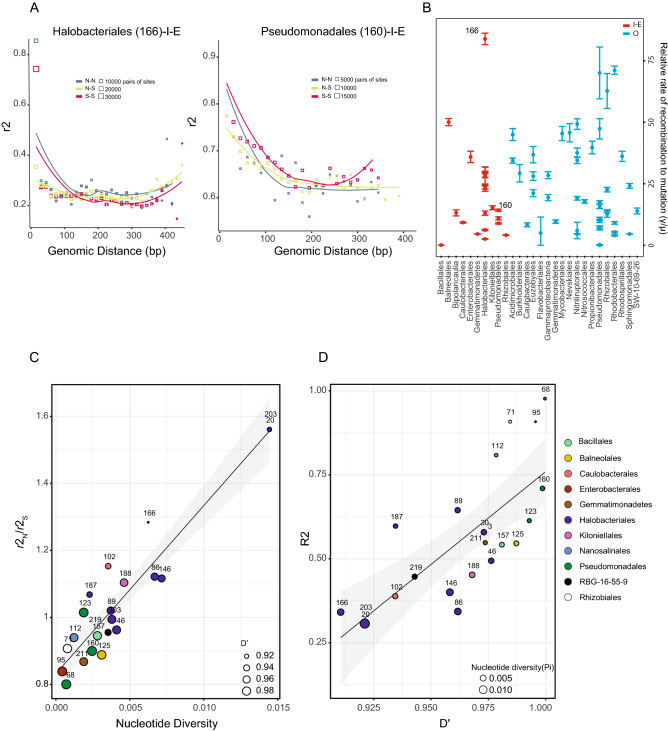
Varying rate of linkage disequilibrium within populations. (**A**) Example of linkage decay represented with (r2) for a pair of loci within population for the highest nucleotide diversity for *Halobacteriales* (166) (left panel), and for the lowest nucleotide diversity for *Pseudomonadales* (160) (right panel) on the inner sabkha. Squares are average of pairs of biallelic sites at that distance that went into the mean calculation. The predicted mutational functions of the paired SNPs are binned by nonsynonymous (N) and synonymous (S). (**B**) Relative rate of recombination to mutation calculated with mcorr package for different population of inner and outer sabkha. *Halobacteriales* (166) and *Pseudomonadales* (160) are labeled on the plot on the inner sabkha. Error bars are 95% confidence interval across 1000 bootstraps. The relative decay of linkage disequilibrium for different populations from inner and outer is summarized in Supplementary Fig. 13. (**C**) The correlation between mean linkages of nonsynonymous-nonsynonymous over synonymous-synonymous (r2N/r2S) and mean nucleotide diversity for pairs of mutations across different species for inner sabkha. D′ is represented by point with different sizes, which is represented by the mean. A linear regression is plotted (Supplementary Fig. 14). (**D**) The correlation between mean r2 to mean D′ across the different population on inner sabkha. A linear regression plot is shown (Supplementary Fig. 15). Correlation of r2N/r2S and nucleotide diversity as well as r2 and D′ are shown in Supplementary Figs. 14, 15.

Measuring linkage disequilibrium (LD) of SNPs spanned by paired end reads^48,55^ in different bacterial population can tell us on the impact of homologous recombination and its effect on genetic diversity. Our results are consistent with the expectation that natural microbial population experience a large amount of recombination, and this is observed with linkage disequilibrium (r^2^) that decay as the distance increases between two SNPs, both inside and outside the sabkha (Fig. 7A, Supplementary Fig. 13). The estimate of the ratio of the neutral rate of recombination to mutation in synonymous third position (Fig. 7B) shows a range for different species on the inside and outside of the sabkha. High rates of recombination is observed in some *Halobacteriales*, *Balneolales*, *Enterobacteriales*, *Pseudomonadales*, *Rhizobiales* and *Rhodobacteriales* species. On other hand, we observe species like Bacillales on the inside and some Pseudomonadales on the outside with very low rates of recombination (Fig. 7B). We also note, the ratio of recombination of nonsynonymous to synonymous (r2N/r2S) increases with higher diversity population (Fig. 7C; Supplementary Fig. 14).

We also measured the LD measure, namely, D′, which is less than 1 suggesting all possible combinations of a pair of biallelic sites, an serves as indication that recombination is present. Our results for inside the sabkha shows that D′ is correlated with mean r^2^ (R^2^ = 0.78, p = 0.005) (Fig. 7D); this correlation is similarly found outside the sabkha (Supplementary Fig. 15). D′ was less than 1 in 9–21% of all site pairs in inside sabkha populations, while D′ < 1 in 2–12% outside the sabkha (Supplementary Fig. 16; Supplementary Table 7). Given that D′ less than one is observed in a significant fraction of loci, as well as the decay of linkage with genomic distance suggests that homologous recombination in the sabkha microbiome.

### Population dynamics and gene-specific selective sweeps across salinity gradient of sabkha

**Figure 8:**
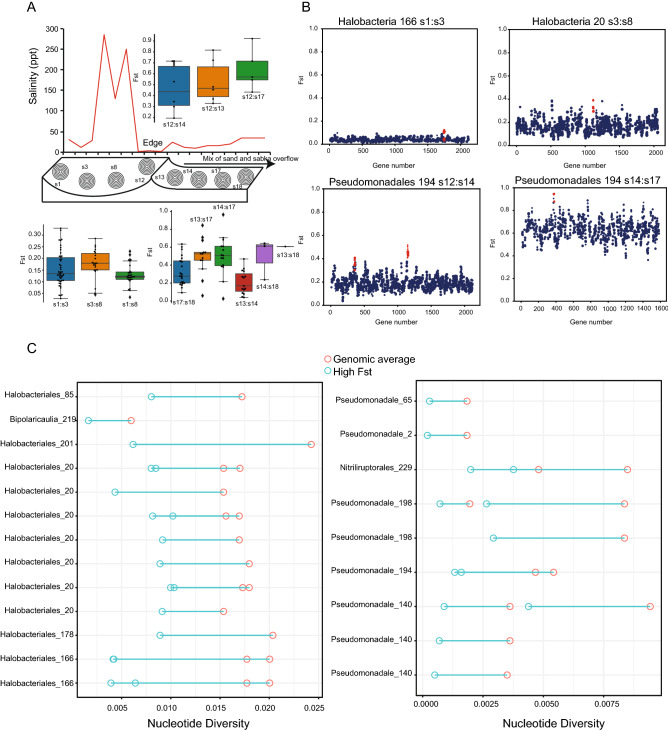
Population differentiation and gene selective sweep analysis. (**A**) Pairwise comparison of population differentiation (Fst) across the inner and outer sabkha along a salinity gradient (top panel). (**B**) Examples of Highly differentiated loci in inner and outer sabkha as well as across the edge. Plot of Fst for genes across the genomes of *Halobacteriale* and *Pseudomonadale* populations on the inner and edge/outside. The distribution of genes is represented by different points, as well as the size of the point is the number of SNPs for that gene. High Fst loci compared to the background are labeled in red. (**C**) Nucleotide diversity for the highest Fst loci (red circle) compared to the average genome (empty circle) for inner (left panel) and outer sabkha (right panel). Summary of gene selective sweep for some population is summarized in Supplementary Fig. 18.

Changes in factors such as soil geochemistry, particle structure, salinity, and many other cues make soil ecosystems heterogeneous, as these factors can change over millimeter scales^56^. Given the complexity in examining the effect of changing abiotic environmental factors over small spatial scales, we estimated the pairwise fixation index Fst for each gene between inside sites (s1, s3, s8, s12) and outside sites (s13, s14, s17, s18); these display a gradual decrease in salinity between sabkha sites (Fig. 8A). Our results show that Fst per populations between sites increases as salinity increase among sites (Fig. 8A), consistent with geographic population structure at most loci across the genome (Supplementary Fig. 17). In contrast, a fraction of Fst values were low (< 5%), consistent with dispersal of alleles among sites.

Population-specific selective pressures acting on specific loci have a signature of high Fst compared to the average genome background^57^. We identified loci with high Fst using a sliding window of 5 genes and tested if these regions had Fst values greater than 1.5 standard deviations above the mean genome average (Supplementary Table 8). The length of genomic region with high Fst was defined when the mean Fst dropped below this threshold. We identified 72 loci with high Fst, in different microbial genomes (e.g., *Halobacteriales* 166 s1 vs. s3) despite having low average Fst values (Fig. 8B, Supplementary Fig. 18).

However, the low nonsynonymous to synonymous ratios in these genes that suggests a signal of purifying selection (Supplementary Fig. 19), as well as the reduction in sequencing coverage at some genomic regions, are both correlated with nucleotide diversity (Supplementary Fig. 20). This suggests that we cannot rely solely on identifying selective sweeps by a significant reduction in nucleotide diversity (Fig. 8C), therefore we looked for a significant increase in LD at these loci compared to the genomic background. Using this criterion, we identified 18 loci with an observed significant reduction in nucleotide diversity and increased LD for different population (*Halobacteriales*, *Pseudomonadales*, *Nitriliruptorales*, *Bipolaricaulia*) within inner and outside sabkha sites (Fig. 8C, Supplementary Fig. 21) suggesting selective sweep events at these genes at different sites in sabkhas. Several of these genes are related to uptake, movement and transport while others have functions related to abiotic stress sensing and response regulation (Supplementary Table 9). Selective sweeps are also evident between sabkha edge and outside sabkha sites (Fig. 8B) (e.g., Pseudomonadales 194), which together suggests that selection is driving some of the differences in microbial population structure across this ecosystem.

### Diverse metabolic potential of microbial communities of sabkha

We investigated the ability of microbial communities of sabkha in harboring specific metabolic activity, and nutrient cycling (e.g.; carbon cycle, Nitrogen cycle, sulfur cycle) (Supplementary Fig. 22). We found, Perchlorate reduction and Chlorite reduction which are enriched on the inside of sabkha (Supplementary Table 10). Interestingly, when we look at the number of genes for combined metabolic pathway; on the inside extreme Halophilic microbes (e.g., *Halobacteriales*, *Cyanobacteriales*) have more than 50 percent of genes (*t* test p < 0.05), while outside, moderately halophilic microbes (e.g., *Pseudomonadales*, *Rhizobiales*, *Nitriliruptorales*) have them (*t* test p < 0.05) (Supplementary Fig. 6, Supplementary Tables 10, 11).

We found evidence for multiple carbon fixation pathways on the inside sabkha, including the Calvin–Benson–Bassham (CBB) cycle in *Cyanobacteriales*, *Halobacteriales*, *Kiloniellales* and *Thiohalorhabdales*, the 3-hydroxypropionate bicycle (3HP bicycle) in *Enterobacterales* and *Pseudomonadales* and the reductive citric acid cycle (rTCA) only found in *Nanosalinales*. In addition to the latter, carbon fixation pathways that are found in most of the outside metagenomes, the outside of sabkha harbors 3HP/4HB cycle, which is only found in *Acidimicrobiales*, *Burkholderiales*, *Nitriliruptorales* and *Polyangiales* (Supplementary Tables 10, 11, Supplementary Fig. 7). The key enzyme methyl-coenzyme M reductase (*mcr*) for methanogenesis was absent from sabkha (Supplementary Tables 10, 11, Supplementary Fig. 7). However, methanotrophy was present through soluble methane monooxygenase (*mmoB*) gene in *Halobacteriales* and *Pseudomonadales* from inside and in *Pseudomonadales, Mycobacteriales,* and *Solirubrobacterales* from outside. We see three abundant genes encoding aerobic monoxide dehydrogenase (*coxSML*) on the inside of sabkha and found only in our *Halobacteriales*, *Kiloniellales and Bipolaricaulia*, whereas it is only in *Rhizobiales*, *Rhodobacteriales* and *Nitriliruptorales* from outside (Supplementary Tables 10, 11).

In term of Nitrogen cycling, we did not observe any metagenomic evidence for nitrogen fixation for inside or outside sabkha. On the other hand, we found evidence for the first step of nitrification and denitrification process, using nitrite oxidoreductase (*nxrAB*), which is involved in nitrite to nitrate and nitrate reductase (*narGH*), for the reverse reaction (Supplementary Tables 10, 11; Supplementary Fig. 7). The second step of denitrification involves 2 genes (*nirSK*, *norBC*) and *nirK* is much abundant on the inside in *Halobacteriales*, *Kiloniellales and Bipolaricaulia* than *nirS*. *nirK* genes are found only in 6 metagenomes from the outside, such as in *Rhodobacteriales, Pseudomonadales.* As for the last step of dissimilatory nitrate reduction to ammonia mediated by nitrite reductase, we see abundance of genes encoding the large and small subunit of the NADH-dependent for of the enzyme (*nirB*, *nirD*) on the outside of sabkha and less abundant on the inside and this shift is the same genes encoding the cytochrome c for of the enzyme (*nrfAH*) (Supplementary Tables 10, 11, Supplementary Fig. 7).

In terms of Sulfur cycling, genes encoding sulfide-quinone reductase (*sqr*) are not abundant in Sabkha, with 3 genomes from the inside (*Cyanobacteriales*, *Enterobacteriales* and *Pseudomonadales*) and 3 genomes on the outside (*Nitrosococcales*, *Gammaproteobacteria*, *Enterobacteriales*). Instead, others genes such as Sulfur dioxygenase (*sdo*) and Sulfate adenylyltransferase (*sat*) are equally abundant on both sides of sabkha. Genes associated with thiosulfate oxidation (soxBY) were most abundant in the outer sabkha and included in metagenomes such as *Burkholderiales*, *Rhizobiales*, *Rhodobacteriales* and *Rhodospirillales*. The inside has only soxY and soxB genes in *Thiohalorhabdales* and *Kiloniellales* respectively (Supplementary Tables 10, 11; Supplementary Fig. 7)*.*

We see evidence for aromatic compounds, complex carbon and fatty acid biodegradation pathways for bacteria and archaea metagenomes. Other pathways, such Arsenic cycling, Urea utilization, oxidative phosphorylation, fermentation and Amino acid utilization are metabolic functions that sabkha ecosystem is harboring (Supplementary Tables 10, 11).

## Discussion

Our results from a UAE coastal sabkha using 16S and whole metagenome analysis shows a shift in archaeal and bacterial taxa composition, with unexpected diversity inside compared to outside the sabkha. The shift of total microbial genetic composition inside vs. outside coastal sabkhas (Figs. 2A,B, 3B, 4C,D), suggests that salinity may act as an important driver of metabolic microbial activity. There is an increased number of halophiles (e.g., *Halobacteriales, Cyanobacteriales*) in the hypersaline (brine > 200 ppt) conditions within a sabkha compared to the less saline environment (brackish < 35 ppt sea water) outside the sabkha where other microbes exists (e.g., *Pseudomonadales*, *Rhizobiales, Nitriliruptorales*) (Supplementary Tables 10, 11, Supplementary Fig. 6, Fig. 4D). This result is consistent with the replication index Irep across all assembled metagenomes where we observed more replication on the inside (> 1.7) compared to outside for the order taxonomic level (Fig. 4E).

The significant biogeochemical consequences can be classified for inside sabkha to sensing/interaction, osmotic stress tolerance and photosynthesis, while outside mostly are involved in osmotic tolerance. Although they have a limited influx of freshwater and little to no surface vegetation, sabkhas have a high potential for diverse carbon sequestration (e.g., CBB cycle RuBisCo pathway as well as others). In our study, we observed *Halobacteriales*, *Cyanobacteriales*, and *Pseudomonadales* taxonomic order from inside sabkha that are likely to contribute to the carbon sequestration of the coastal sabkha (Supplementary Tables 10, 11). Cyanobacteriales species are at the heart of light-driven metabolic processes in sabkhas; we note a high abundance of *Chloroflexi* in our samples, similar to the study carried out by Ward et al.^58^. The cyanobacteria, *Rubidibacter* from inside sabkha have rod-like morphology that is similar to *Synechocystis*^59^. It was discovered that betaine is synthesized in cyanobacteria, through Choline as a precursor and its concentration is salt dependent in halophilic bacteria^60,61^. Betaine is a common osmoprotectants in halotolerant and halophilic microorganisms^61,62^ and an important feature of halotolerant Cyanobacteria as well as phototrophic bacteria^63^. The release of high concentrations of homoserine betaine upon lysing likely impacts the biogeochemical cycling of carbon and nitrogen in oligotrophic subtropical and tropical oceans^64^. *Rubidibacter* species may function as solar umbrellas that enhance the viability of more radiation-susceptible microbes, through the accumulation of the UV-blocking pigments zeaxanthin, beta-carotene, and echinenone to high levels^59^. A similar hypothesis was made by Di Loretta et al.^65^ in their description of filamentous anoxygenic photosynthetic bacteria (FAPB) mats and their dominance of the uppermost photo-oxic layers in the Khor al-Adaid sabkha in Qatar^65^. Thus, large-scale interdependence of cyanobacteria in juxtaposition with other microbial assemblages may be a crucial factor in thriving, dynamic sabkha development.

Interestingly, in addition to the light harvesting photosynthetic cyanobacteria, Halophitic Archaea (e.g., *Halobacteriales*, *Nanosalinales*) found inside sabkha expand the diversity of known Carboxydovores (CO-oxidizers). These CO-oxidizing groups have the ability to use light-driven proton pump bacteriorhodopsin to supply energy^66^. Different mechanisms such as CO-oxidation, light-harvesting complexes to sequester energy is very important for heterotrophs inside sabkha subjected to extreme saline environment, which impose extra costs for osmoregulation. This is also consistent with the noticeable presence of perchlorate reduction on the inside more than outside as well as more Chlorite reduction for these groups, which is an evidence for plausible microbe reaction on Martian brines and the reaction will emit oxygen, which will strongly contribute to facilitating the process of Mars colonization^66,67^.

Consistent with the CO-oxidizers, nitrogen cycling via denitrification (nitrate to nitrite) is an abundant portion of the inside (*Halobacteriales*, *Nanosalinales*) and outside sabkha (*Pseudomonadales*, *Rhizobiales*), which is consistent with other studies^68,69^. This abundance of denitrification genes contrasts with the absence of nitrogen fixation genes (Supplementary Fig. 7). For instance, Cyanobacteria are known nitrogen-fixing bacteria^70^, however, they have only *norB* genes, which is required for nitric oxide reduction during denitrification. The lack of nitrogenase sequences could be the lack of more sampling of deeper anoxic sediments.

The presence of a dynamic sulfur cycle via sulfide oxidation is prominent in inside and outside sabkha. This process takes place via aerobic and anaerobic pathways, similar to the denitrification process, using alternative electron acceptors^71^. The sulfide quinone reductase (sqr) is known to be involved in energy conservation, where it is linked to ATP production via sulfide oxidation or sulfide detoxification, where the sulfide oxidation is only used to protect microbial cell from damage^72–74^.

Other strategy for carbon fixation is via methylated carbon source in methanogenesis, which is a known strategy to overcome competition for CO_2_ in saline environment^75,76^. Sequences encoding hydrogenases for methanogenesis is absent on the inside, and outside sabkha, however Methanotrophy is present in both side of sabkha (Supplementary Fig. 7). The lack of methanogenesis genes may require deeper sediments sampling.

Ribosomal genes, which are in general conserved and subject to purifying selection, show lower diversity across sabkhas; however, we observe a significant increase in the diversity of ribosomal genes in *Halobacteriales* (p < 0.05) (Fig. 5A,B). Interestingly, ribosomal biosynthetic genes LAP and lanthipeptide have the highest diversity inside sabkhas (Fig. 5B; Supplementary Fig. 10) for *Halobacteriales*. LAP and lanthipeptide are known to coordinate cooperative social interaction among bacteria, including toxin secretion, acquisition of metals such as iron for all bacteria and signaling antimicrobials molecules^77–79^.

We observe extensive horizontal gene transfer (HGT) between microbial species in the sabkha environment (Fig. 6A). This process has been demonstrated in the soil microbiome, where gene gain and loss are evidenced by the difference in genome sizes among closely related isolates^80^. Horizontal gene transfer is an important process in sharing beneficial genes across populations and communities. Enrichment PFAM analysis (Fig. 6B) show that horizontally transferred genes related to abiotic stress tolerance, including loci for metabolic/catabolic process, temperature response, detoxification and transport of solutes, lipid modification have higher diversity than the genome average (Supplementary Tables 12, 13). Interestingly, we observed HGT inside sabkhas as a source of social cooperative development genes among the microbiome. This is consistent with what the LAP and lanthipeptide gene we observe in *Halobacteriales*, which is known to be involved in sensing and communicating via an ABC transporter^81^.

The relative rate of recombination and levels of LD confirm that homologous recombination is common in sabkha. We see a large variation in recombination for greater than 90 percent of the populations (Fig. 7B), and a significant fraction of D′ less than 1 with LD decay as a function of distance across the genome. This provides a good evidence for homologous recombination in sabkha populations. Moreover, the increase of the ratio of r2_N_/r2_S_ with nucleotide diversity (Fig. 7C), suggest an increase of the ratio of beneficial to slightly deleterious nonsynonymous SNPs. The presence in different genomes of genes that confer natural competence for DNA uptake and recombination, such as *ComEC* and archaeal *pili IV,* suggest that they are likely to play a role in this process (Supplementary Table 14).

Our results show that a change in salt concentration at short length scales (within centimeters) contributes to divergence of alleles across the sabkha salt gradient. We observed allele frequencies for genes consistent with changes in salinity concentration (Fig. 8A) as shown in Fst comparisons between sites inside and outside the sabkhas (Fig. 8A; Supplementary Fig. 18). Specific loci with high Fst (Fig. 8B) show decreased nucleotide diversity compared to the average background genome (Fig. 8C) as well as high LD compared to the average genome background (Supplementary Fig. 21), consistent with selective sweeps events along the salinity gradient across sabkha. In addition, these genes have higher nonsynonymous to synonymous ratios, suggesting recent selection on beneficial nonsynonymous mutations. The differentially selected genes across sabkha, which includes ABC transporters, abiotic stress genes, quorum sensing in *Halobacteriales*, osmoprotection in *Pseudomonadales* and *Rhizobiales* (Supplementary Table 9), suggest that gene-specific selection is another evolutionary force driving genetic structure in sabkha population. The hydrocarbon-degrading bacteria Proteobacteria, Bacteroidetes, Actinobacteria and Halobacteria have previously been documented in high saline soil environments^82^, and further study of members from these clades may provide insight into novel industrial applications. Therefore, the study of the whole metagenomes assembled genomes and protein-coding sequences of these microbes, and other undiscovered species (cultured and uncultured) through different method of isolation, can provide foundations for new green chemistry applications. Salt stress tolerance genes isolated from Halobacteria, Proteobacteria, Actinobacteria, Gemmatimonadetes, Bacteroidetes, Firmicutes, and Acidobacteria have been previously cloned, and new osmotolerance mechanisms were revealed that might provide benefits for biosaline agriculture^83^. We managed to isolate 12 species from inside and outside sabkha, and some important physiological traits have been measured and are ready for more detailed functional studies related to biosaline agriculture applications (Supplementary Fig. 23, Supplementary Table 15).

## Conclusions

This study is the first to examine the sabkha ecosystem not only for its microbial composition using 16S rDNA analysis^13,65^, but with whole metagenomic data from sabkha sites that vary across a salinity gradient. This provides a cohesive picture of the genetic structure of sabkha soil population within a relatively short spatial scale. We conclude that the sabkha environment, both inside this high salinity ecosystem as well as the adjacent environment outside the sabkha, is a dynamic ecosystem, containing eubacterial and archaebacterial species. Interestingly, despite its stressful environment, sabkhas appear to harbor even greater microbial diversity compared to the area outside. Moreover, our metagenomic analysis suggests that some of the evolution in the sabkha environment is driven by the horizontal transfer of genes, as well as recombination and gene-specific selection, which shape the population genetic structure in this ecosystem. In addition, this study shed the light on the importance and potential of these bacterial consortium in alleviating and enhancing abiotic stress for agricultural application.

## Supplementary Information

Supplementary Information.

Supplementary Tables.

## Data Availability

The 16S rDNA (V3–V4) and Whole metagenomic data generated during this study were deposited in NCBI-SRA database under the Bioproject id PRJNA753691. Metagenome resolved assembled genomes are located in https://github.com/KCGEB-UAEU/Sabkha-dataanalysis/tree/main/Bins_genome. Additional information is located at this github (https://github.com/KCGEB-UAEU/Sabkha-data-analysis). SNPs, linkage, diversity, and pairwise Fst comparison for inner and outer sabkha data is hosted at: https://doi.org/10.5281/zenodo.6381683. All horizontal genes transfer between different orders for inner and outer sabkha: https://github.com/KCGEB-UAEU/Sabkha-data-analysis.

## Abbreviations

ANI: Average Nucleotide Identity
AMGs: Auxiliary metabolic genes
HGT: Horizontal gene transfer
OTUs: Operational taxonomic units
LD: Linkage disequilibrium
SNP: Single nucleotide polymorphism
KEGG: Kyoto encyclopedia of genes and genomes
FDR: False discovery rate
TDS: Total dissolved solids
