# Supplementary material for: Salt flat microbial diversity and dynamics across salinity gradient
Source: Sci Rep. 2022 Jul 4;12:11293. doi: 10.1038/s41598-022-15347-8 (PMC9253026; doi:10.1038/s41598-022-15347-8)
Supplement: Supplementary file 1 — Supplementary Information. [file 41598_2022_15347_MOESM1_ESM.pdf]

Supplementary information for manuscript entitled:

Salt flat microbial diversity and dynamics across salinity gradient

Khaled M Hazzouri [1#], Naganeeswaran Sudalaimuthuasari [1#], Esam Eldin Saeed[1], Biduth Kundu[2], Raja Saeed Al-Maskari[2], David Nelson[3], Alya Ali AlShehhi[2], Maryam Abdulla Aldhuhoori[2], Dhabiah Saleh Almutawa[2], Fatema Rashed Alshehhi[2], Jithin Balan[1], Sunil Mundra[2], Mohammad Alam[2], Kourosh Salehi-Ashtiani[3], Michael Purugganan[3, 4] and Khaled MA Amiri[1, 2]*

This supplementary information contains:

Supplementary figures related to the manuscript
Supplementary methods for bacterial isolation from sabkha
References for Supplementary methods.

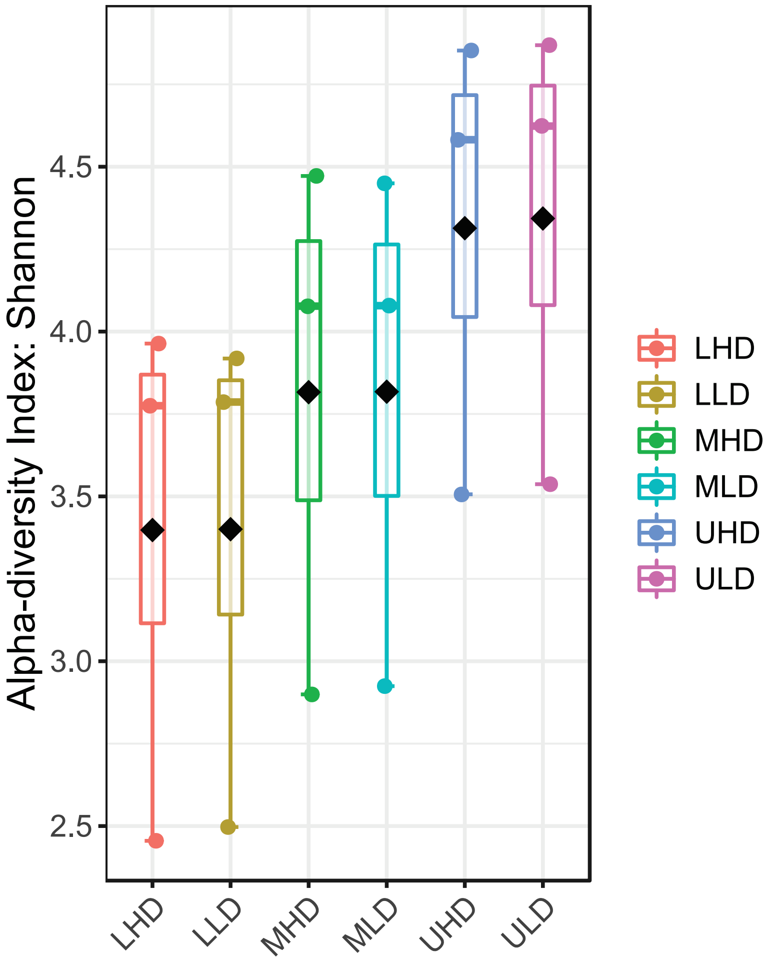

Supplementary Figure 1. Comparaison of Alpha diversity measure at 3cm depth for upper, lower and middle sections across the eighteen 16S samples, and with high (HD) and low depth per section (LD) (UHD/ULD; LHD/LLD; MHD/MLD), from Inside, Edge and Outside (ANOVA F-value=0.86572, p-value=0.53138)

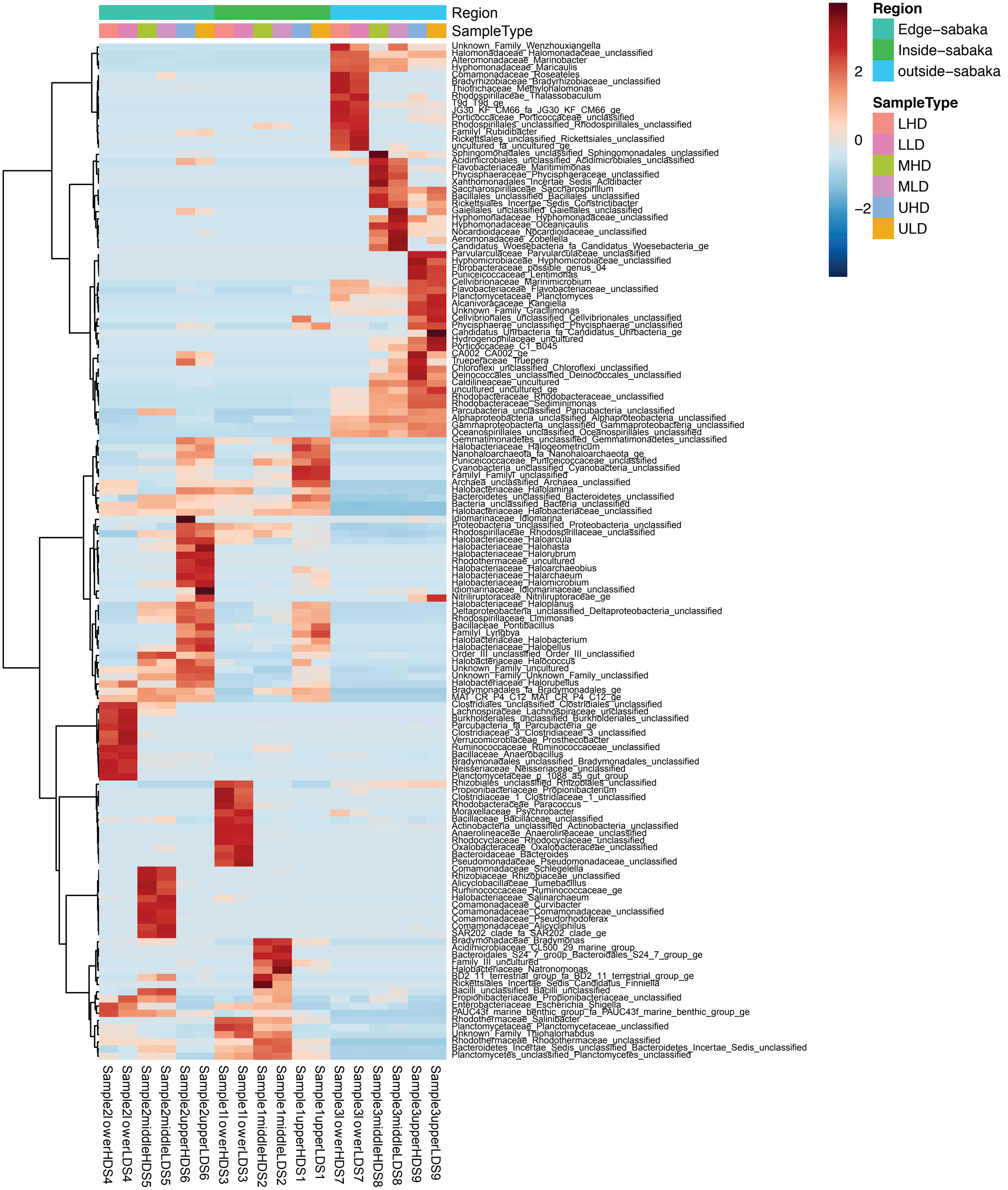

Supplementary Figure 2. Heatmap comparaison of samples type from upper, lower and middle section, with higher (HD) and lower depth (LD) per section at the genus level using the MicrobiomeAnalyst as well as per region for Inside, Edge and Outside Sabkha. The color bar gradient is the logarithm of matrix representing the abundance of microbial feature (OTUs) detected.

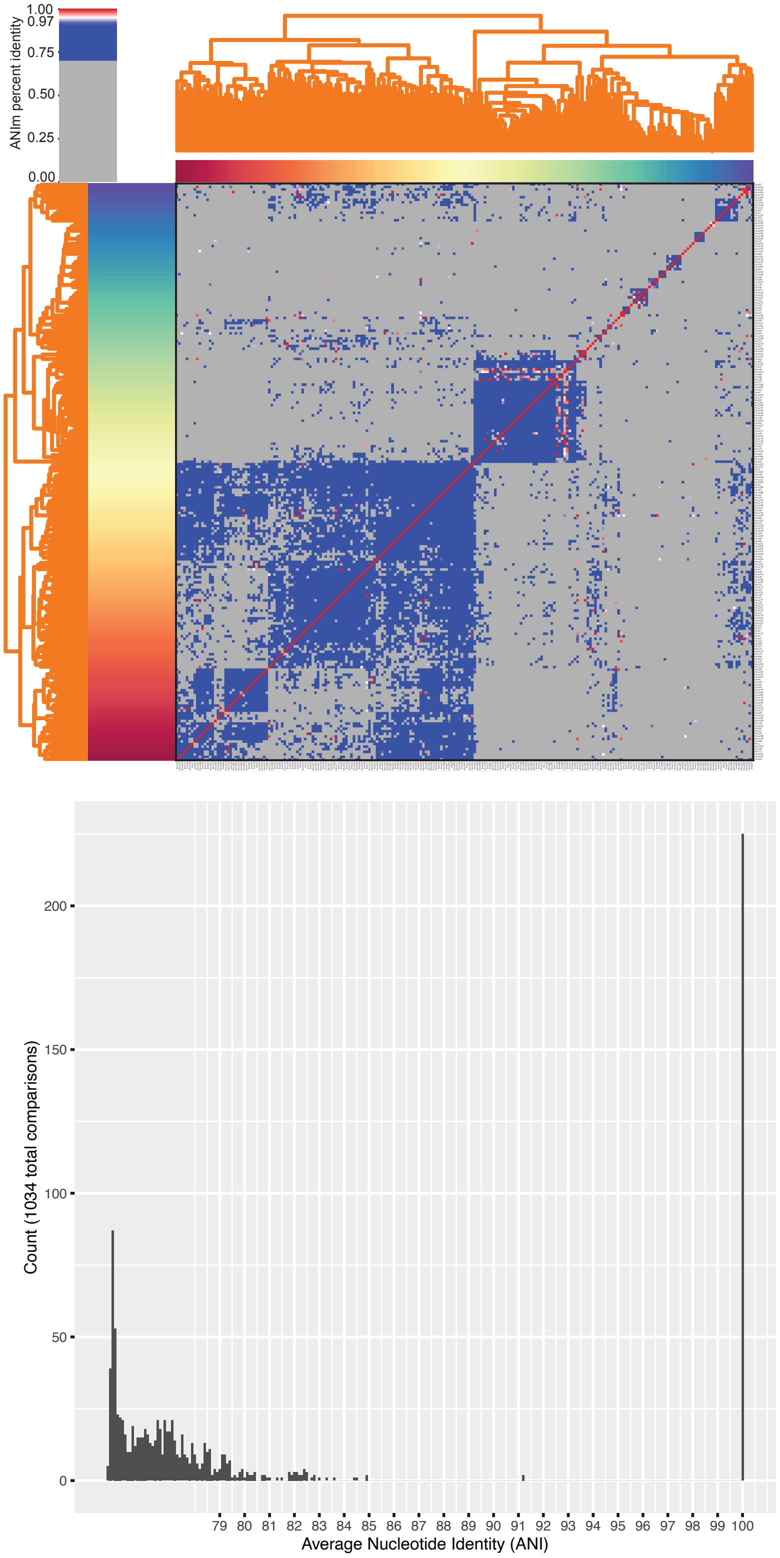

Supplementary Figure 3. Heatmap of ANI percent identity for the 225 bins described genomes. Cells in the heatmap correspond to 97 % ANI sequence identity are coloured in red. Blue cells correspond to genomes that don't belong to the same species. Color intensity fades as the comparaison approach 97 % ANI sequence identity. Colour bar above and left of the heatmap is indicating the source species assignments for each bin in the analysis. Hierarchical clustering was done suing simple linkage of ANI percent identity. The bottom histogram shows the % ANI identidy and counts of MAG for indentical and different genomes.

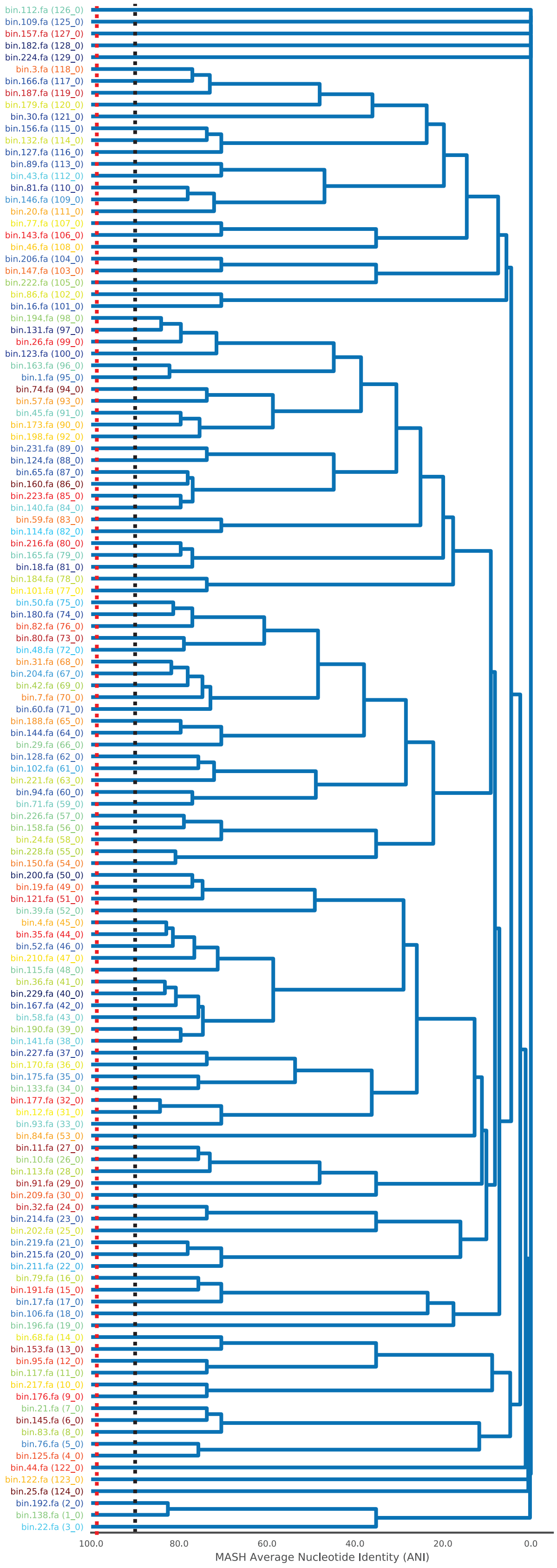

Supplementary Figure 4. Clustering dendogram summarizing the pair-wise MASH distance among the 225 genomes using dRep. The black dotted line depict the primary ANI to create the clus-ters, which is at 90% ANI, and the red one is at secondary ANI 97 %, where similar colors of bins on the left representing the genomes that belong to the same cluster. A total of 129 dereplicated genomes, were generated.

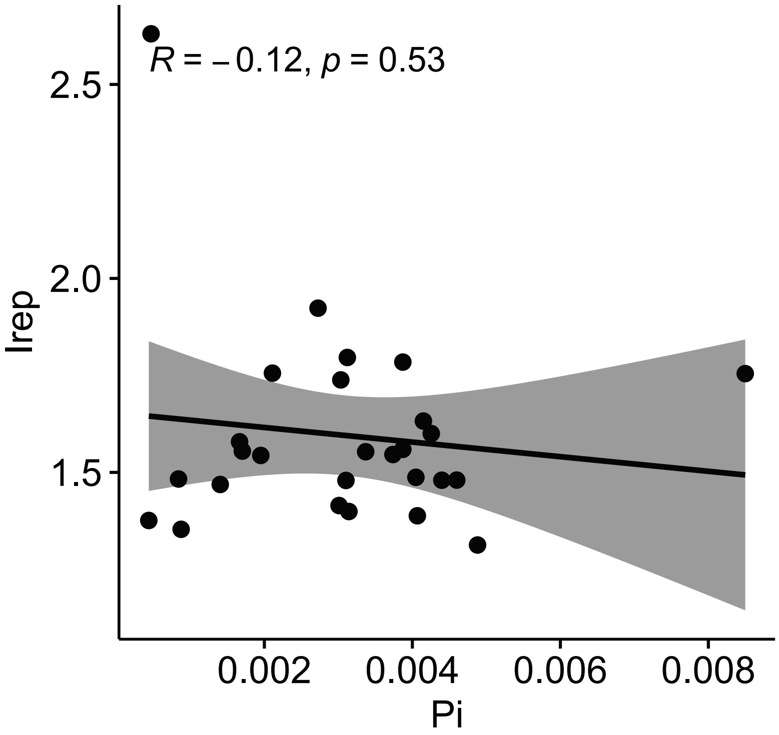

Supplementary Figure 5. Scatterplot is depicting the correlation of the index of replication (Irep) with nucleotide diversity (Pi) among the different phylum. There is no significant correlation (pearson, R=-0.12, p=0.53) between them, suggesting that diversity seen in our sabkha is not correlated with abundance.

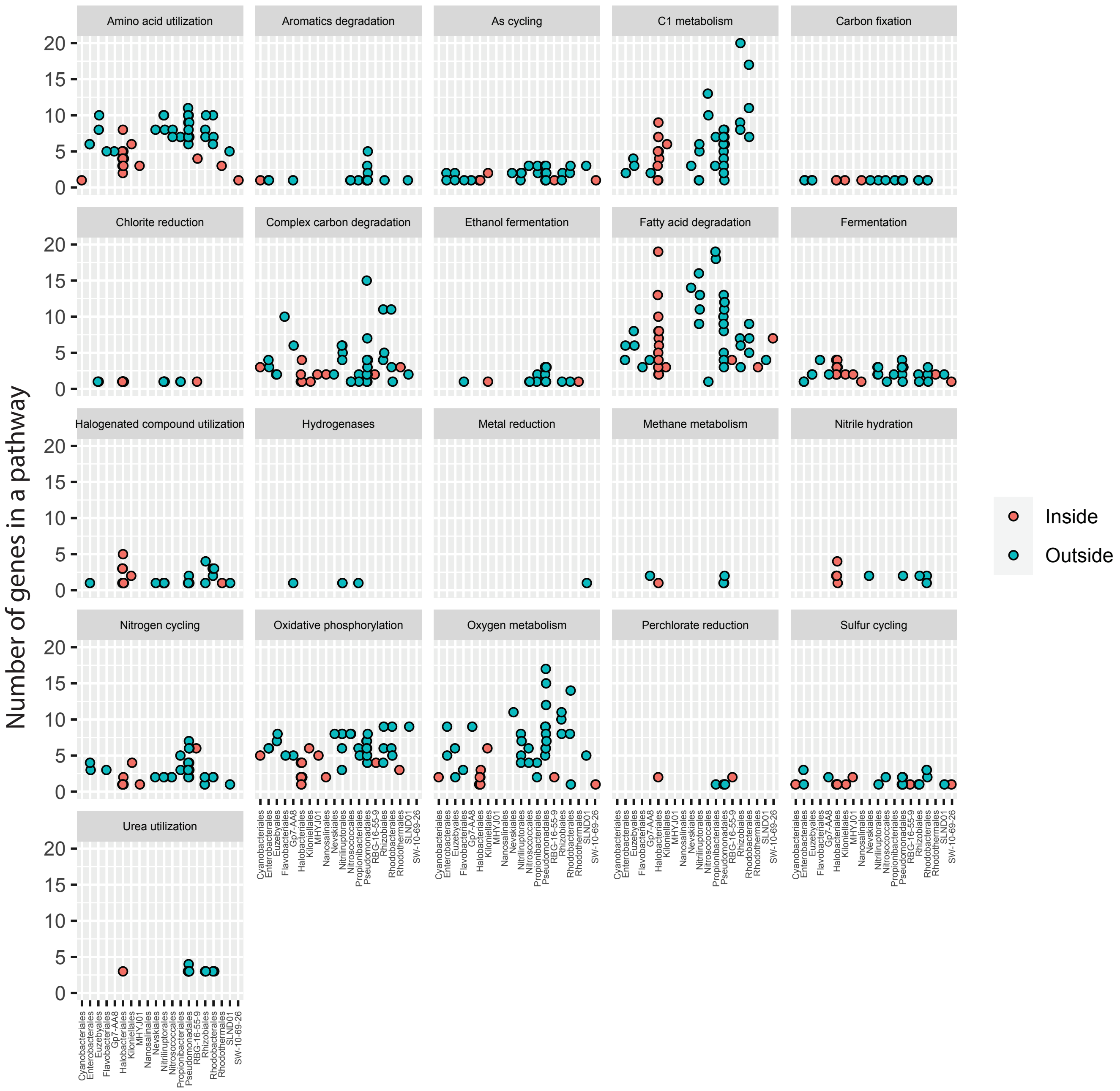

Supplementary Figure 6. Plot of the number of each HMM mapped genes in a different metabolic pathway. The gene numbers across the different taxonomic order highlight the presence of significant abundance of genes for the extreme halophilic Halobacteriales, Cyanobacteriales on the inside, while similar abundance is present on the outside for moderately halophilic Pseudomonadales, Rhizobiales, Nitrilirup-torales (t-test, p <0.05).

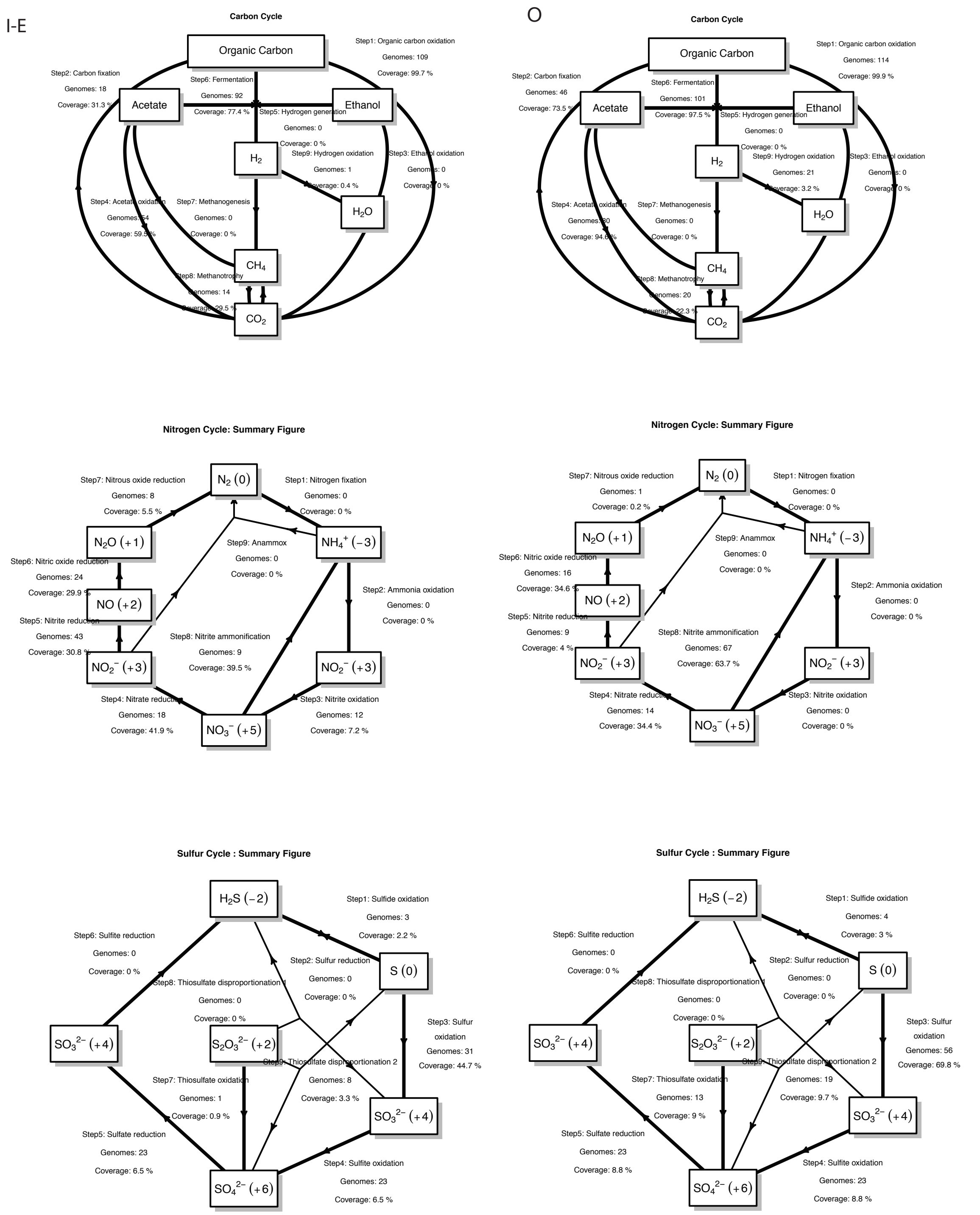

Supplementary Figure 7. Summary diagrams showing the nutrient cycling for carbon fixation, nitrogen cycling and sulfur cycling, as an output of METABOLIC tool. The Diagrams are separated by Inside-Edge (I-E) and outside (O). The number of genomes as well as coverage that has HMM genes into relevant pathways is shown with the direction of the pathway.

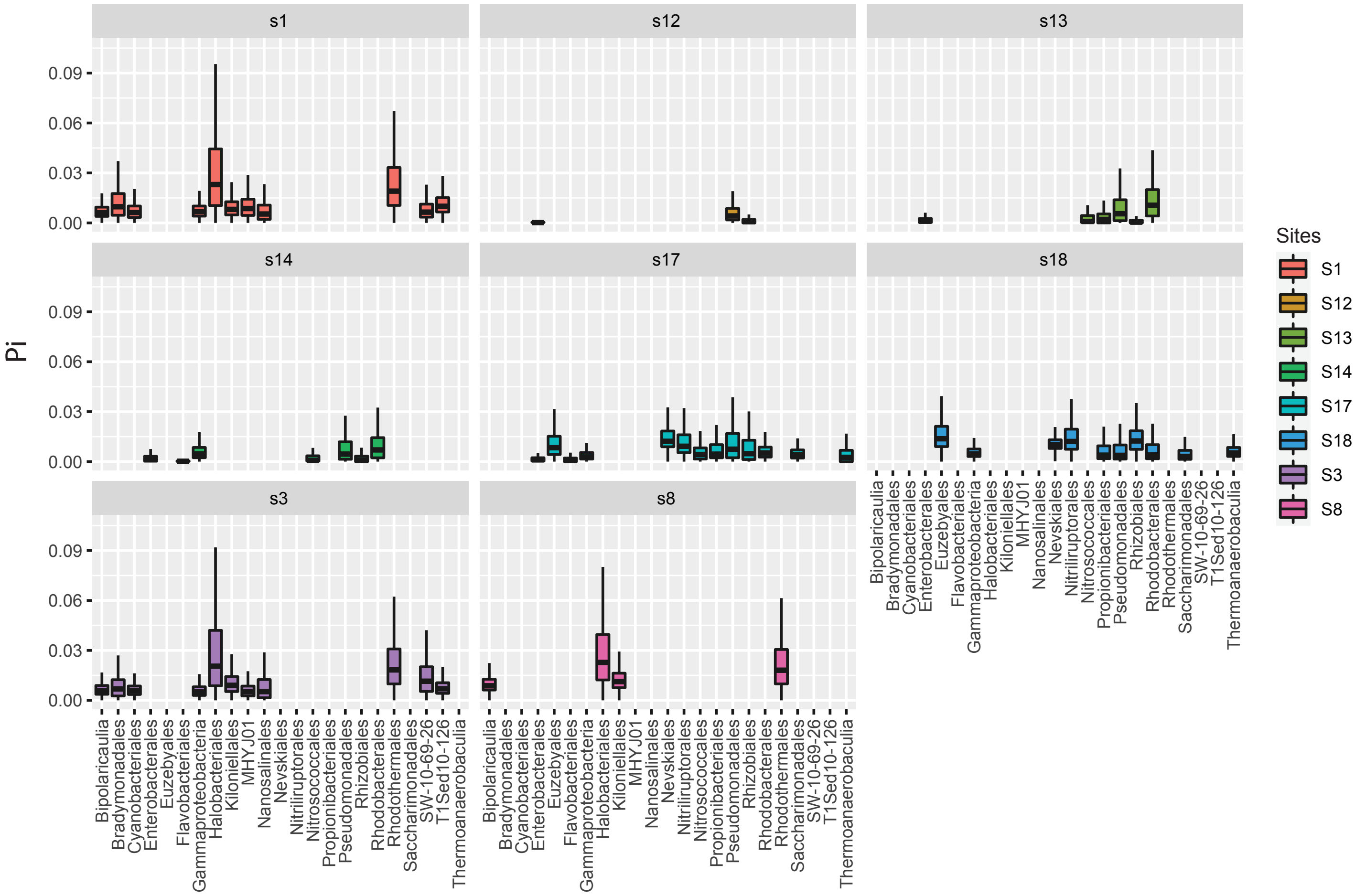

Supplementary Figure 8. Plot showing nucleotide diversity that persist at finer scale per site across the sabkha inside and outside study region. Higher diversity was significant for Halobacteriales and Pseudomonadales populations on the inside and Rhizobiales and Pseudomonadales from outside (pairwise t-test Bonferroni $p < 0.05$).

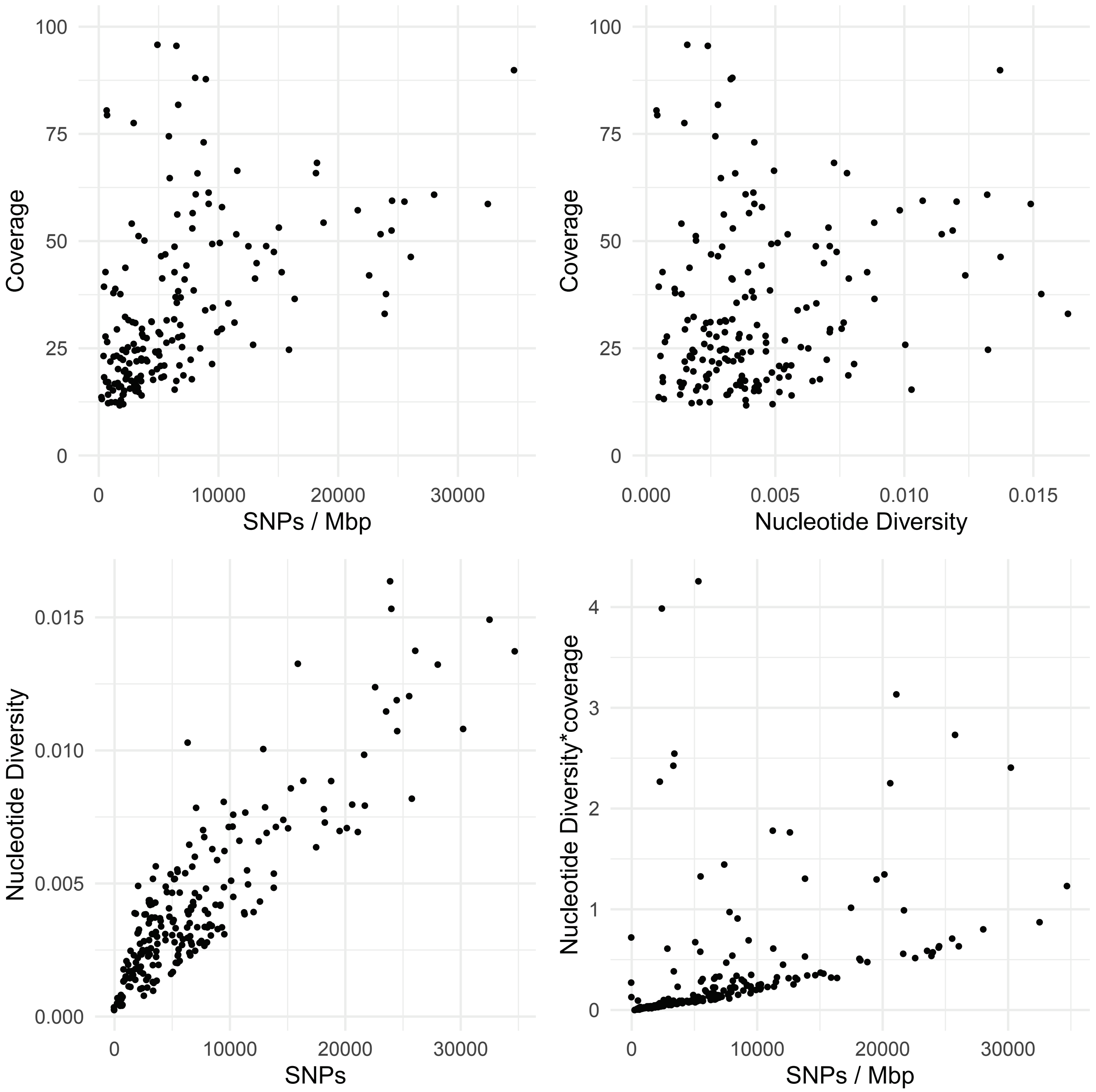

Supplementary Figure 9. Nucleotide diversity was measured using the total number of SNPs/Mbp as metrics methods, which is less sensitive to changes in coverage and is done both for single site and averaged across loci, where also frequencies of SNPs is included not only SNPs by itself.

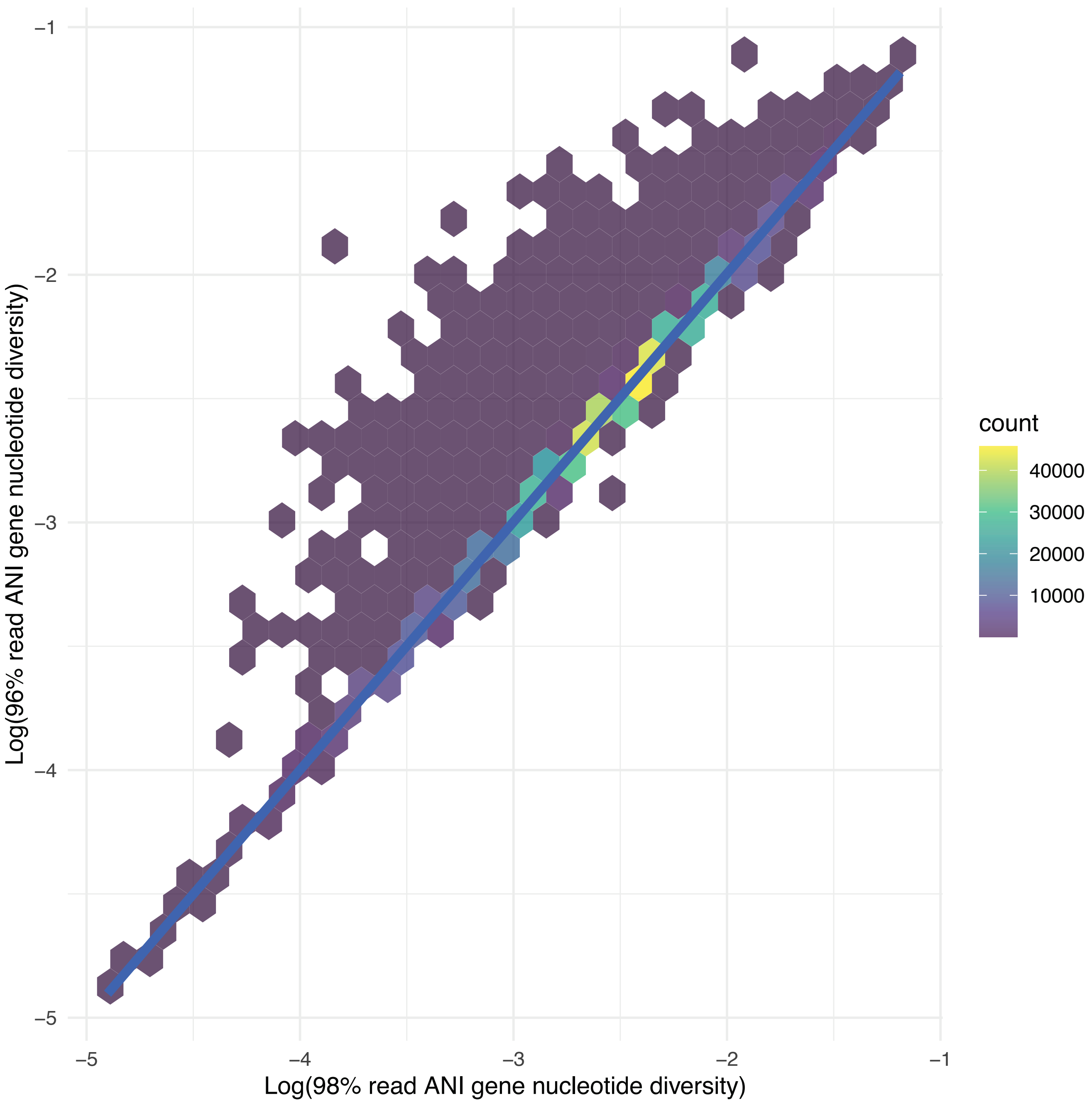

Supplementary Figure 10. Correlation plot of Log (98% read ANI gene nucleotide diversity) versus Log (98% read ANI gene nucleotide diversity) (Pearson R=0.9, $p < 0.05$) is showing comparable gene nucleotide diversity across the different populations per site in the sabkha region.

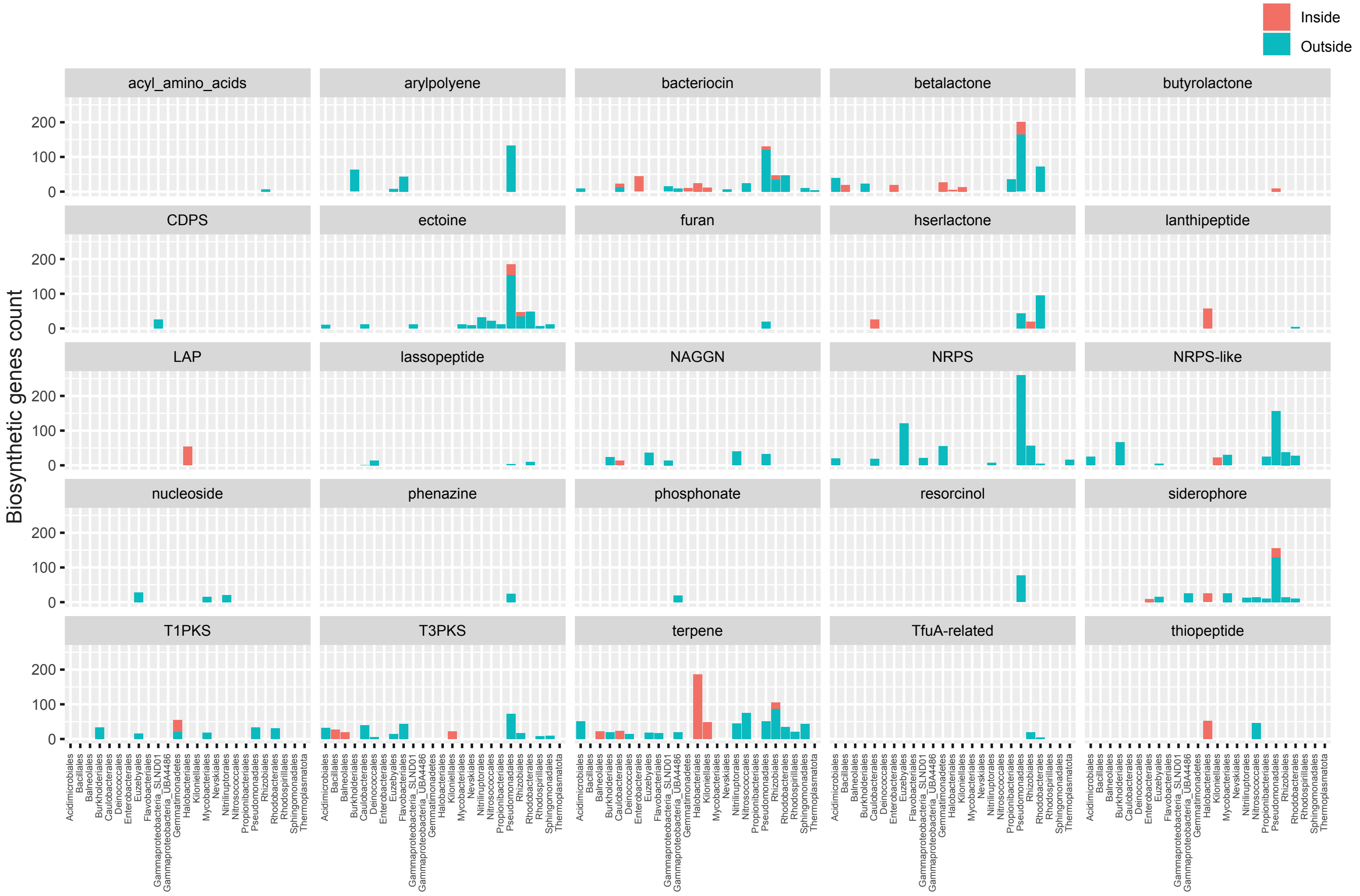

Supplementary Figure 11. The plot of the number of different biosynthetic genes for the different taxonomic order showing unique evolution of biosynthetic gene classes counts to specific population on the inside and outside sabkha.

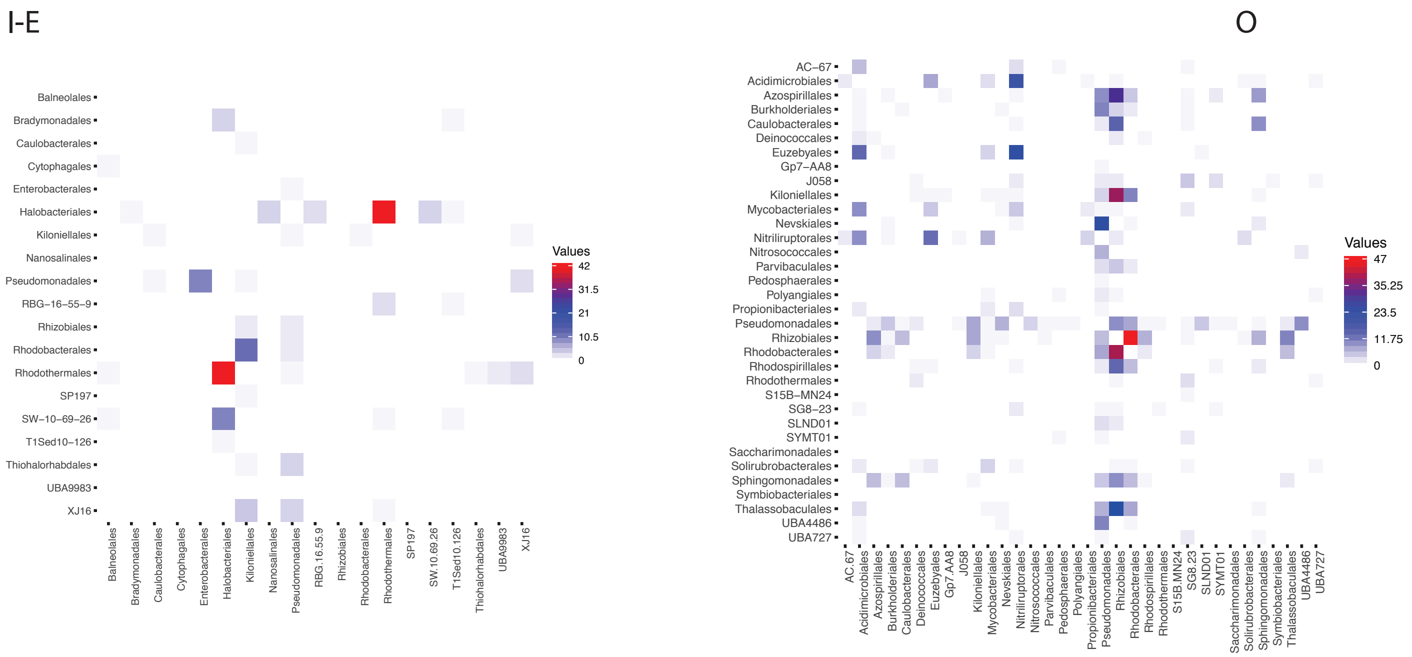

Supplementary Figure 12. Pairwise interactions of Horizontal gene transfer (HGT) across the inside (I-E) and outside (O) using MetaChip. The color bar depicts the number of interaction happening among the taxonomic order level.

Examples of some linkage disequilibrium (r2) decay on inside and outside sabkha

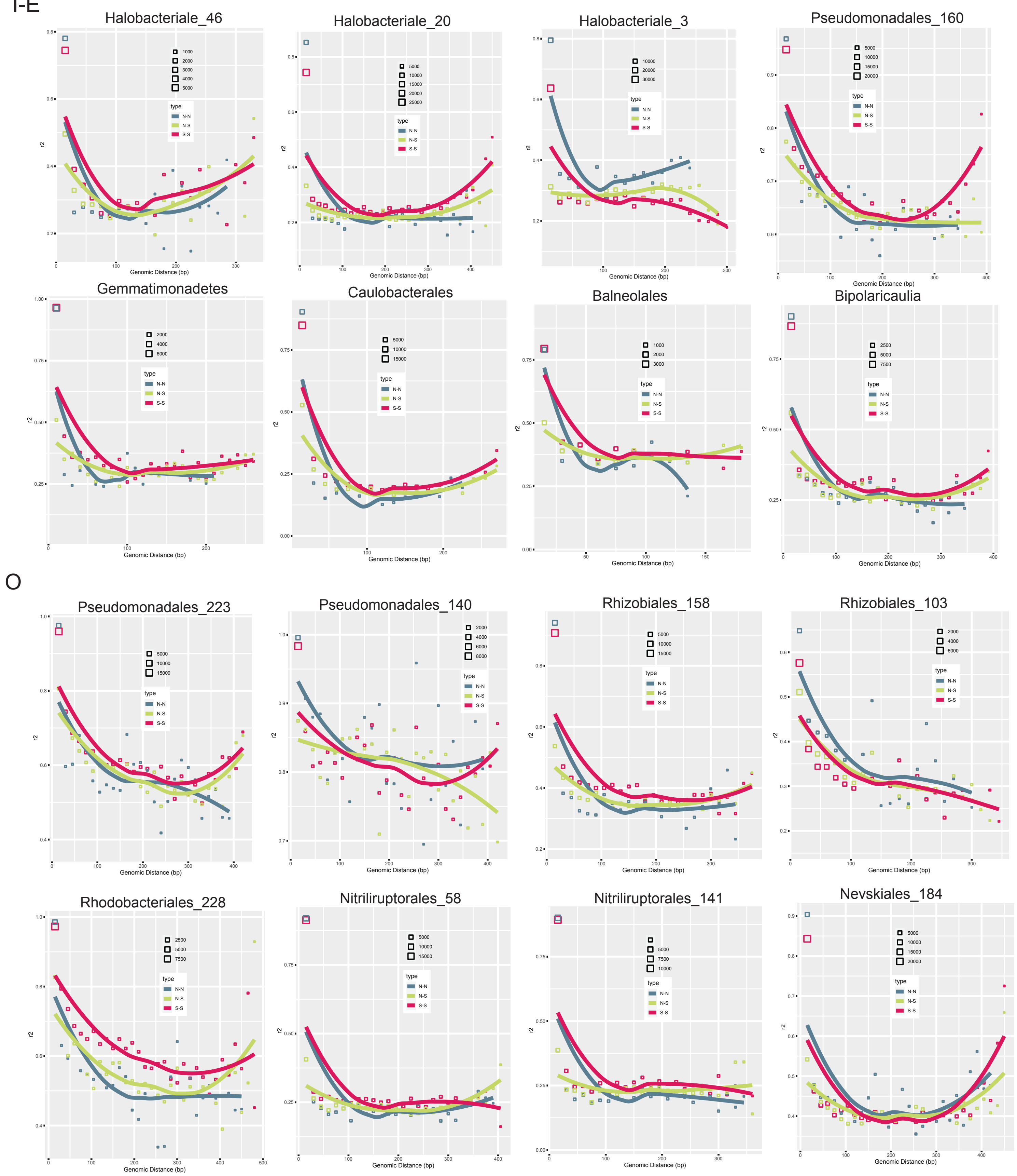

Supplementary Figure 13. Some examples showing linkage disequilibrium (r2) decay with distance for different taxonomic orders for inside (I-E) and outside (O)sabkha.
The type SNPS correlation is depicted as N-N (nonsynonymous-nonsynonymous), S-S (synonymous-synonymous) and N-S (nonsynonymous-synonymous) as well as the varying number sites depicted with squares at different sizes.

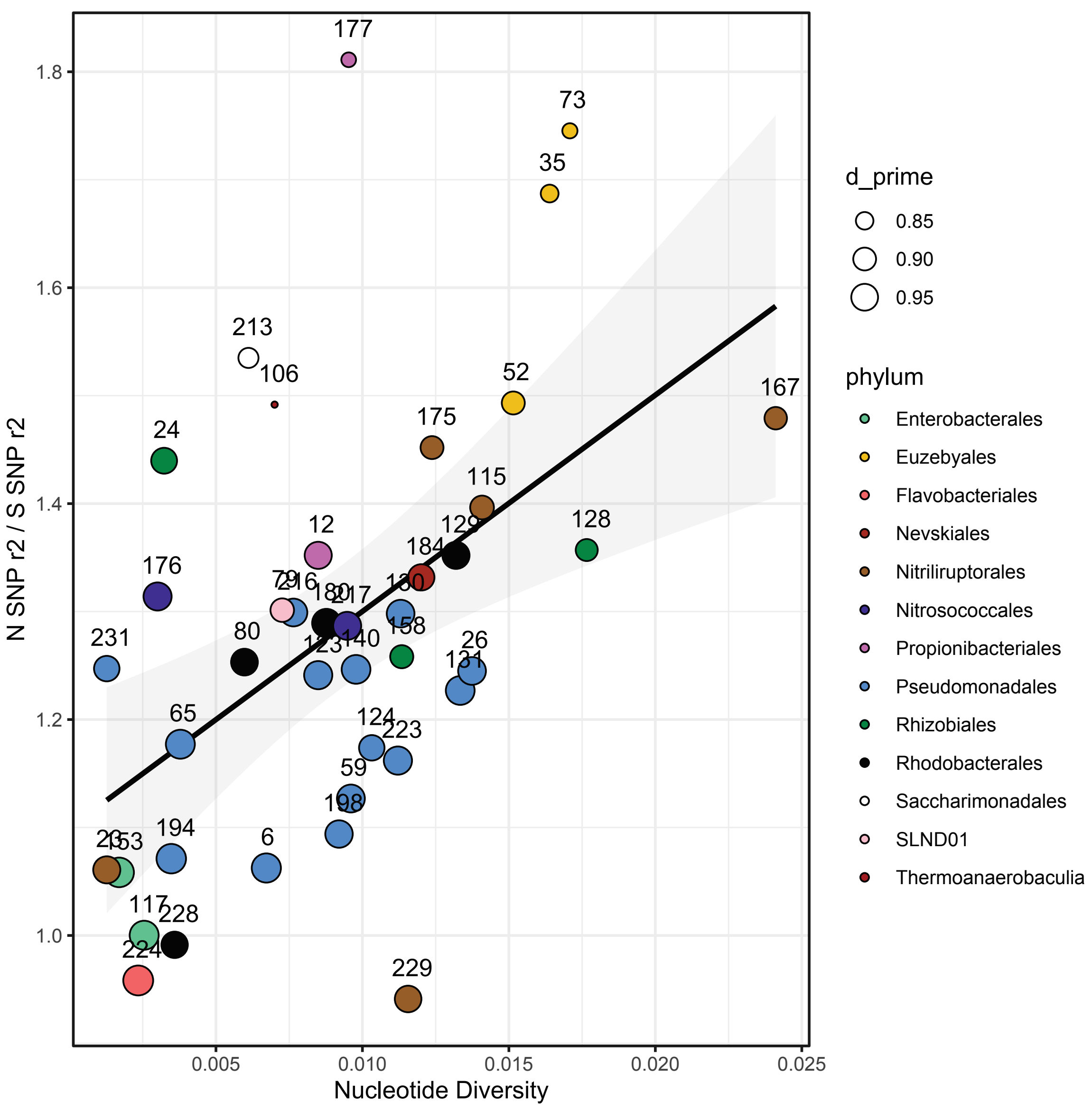

Supplementary Figure 14. Pearson correlation is showing an increase of the r2N/r2S ratio with nucleotide diversity on the outside of sabkha. The different representative genomes are colored accordingly. D' is depicted with circle that vary in size.

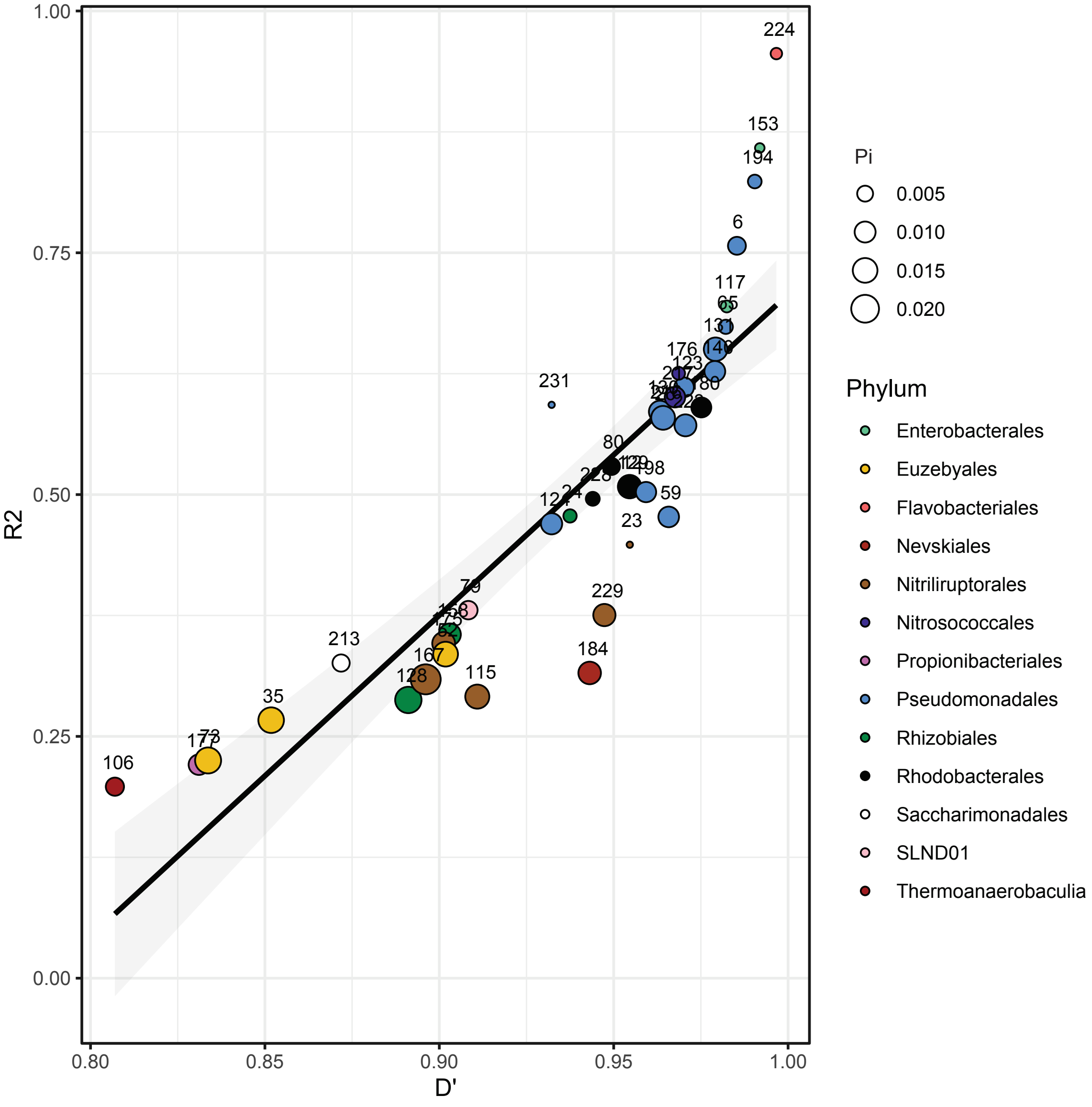

Supplementary Figure 15. Outside sabkha show the average D' for a population is correlated with mean r2. Nucleotide diversity (Pi) is depicted with varying empty circles in size.

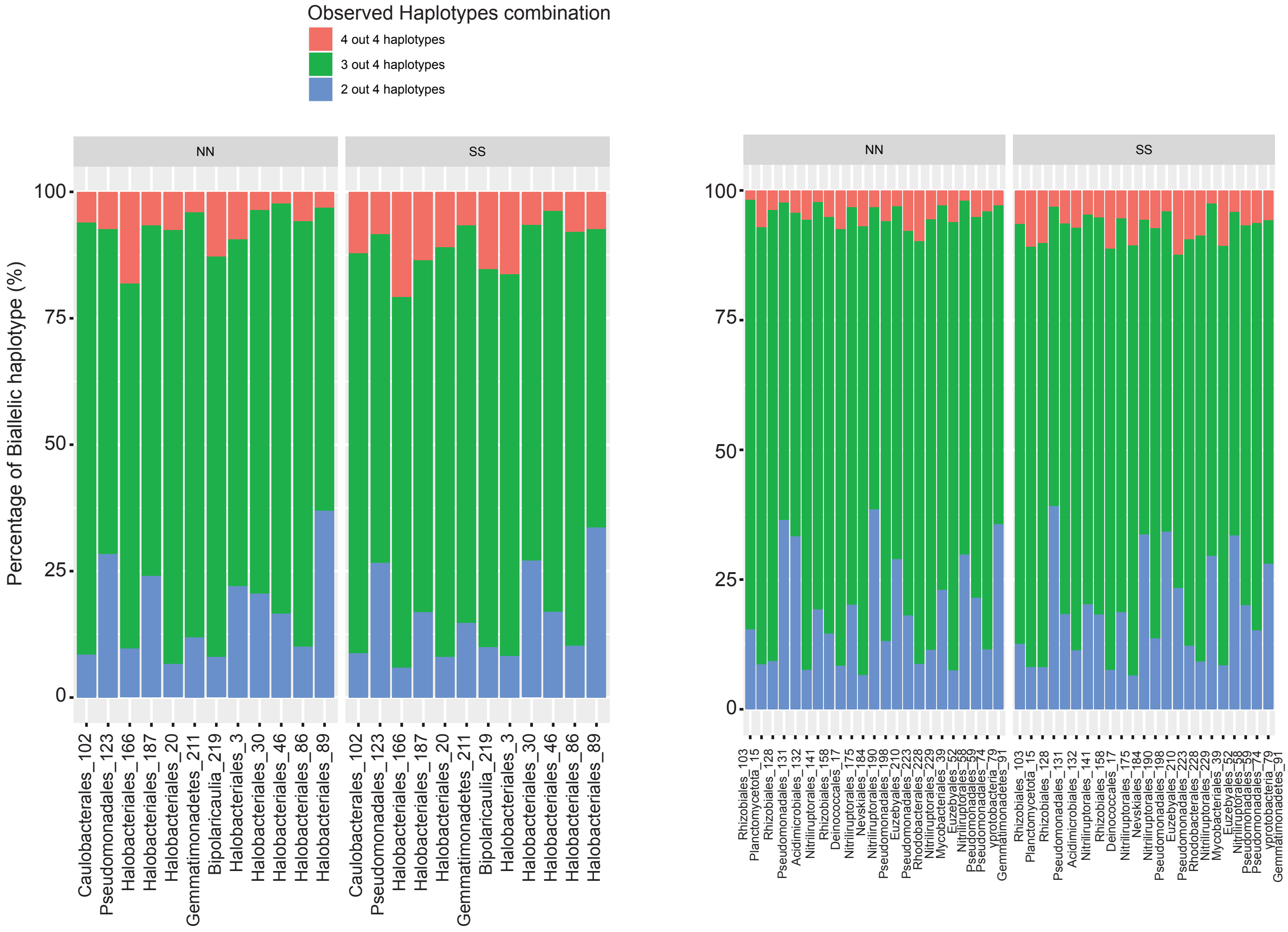

Supplementary figure 16. The observed percentage (%) of biallelic haplotype combination of 4 out 4, 3 out 4 and 2 out 4 for pairs of SNPs within ~1kb for inside (I-E) and outside (O) sabkha populations.

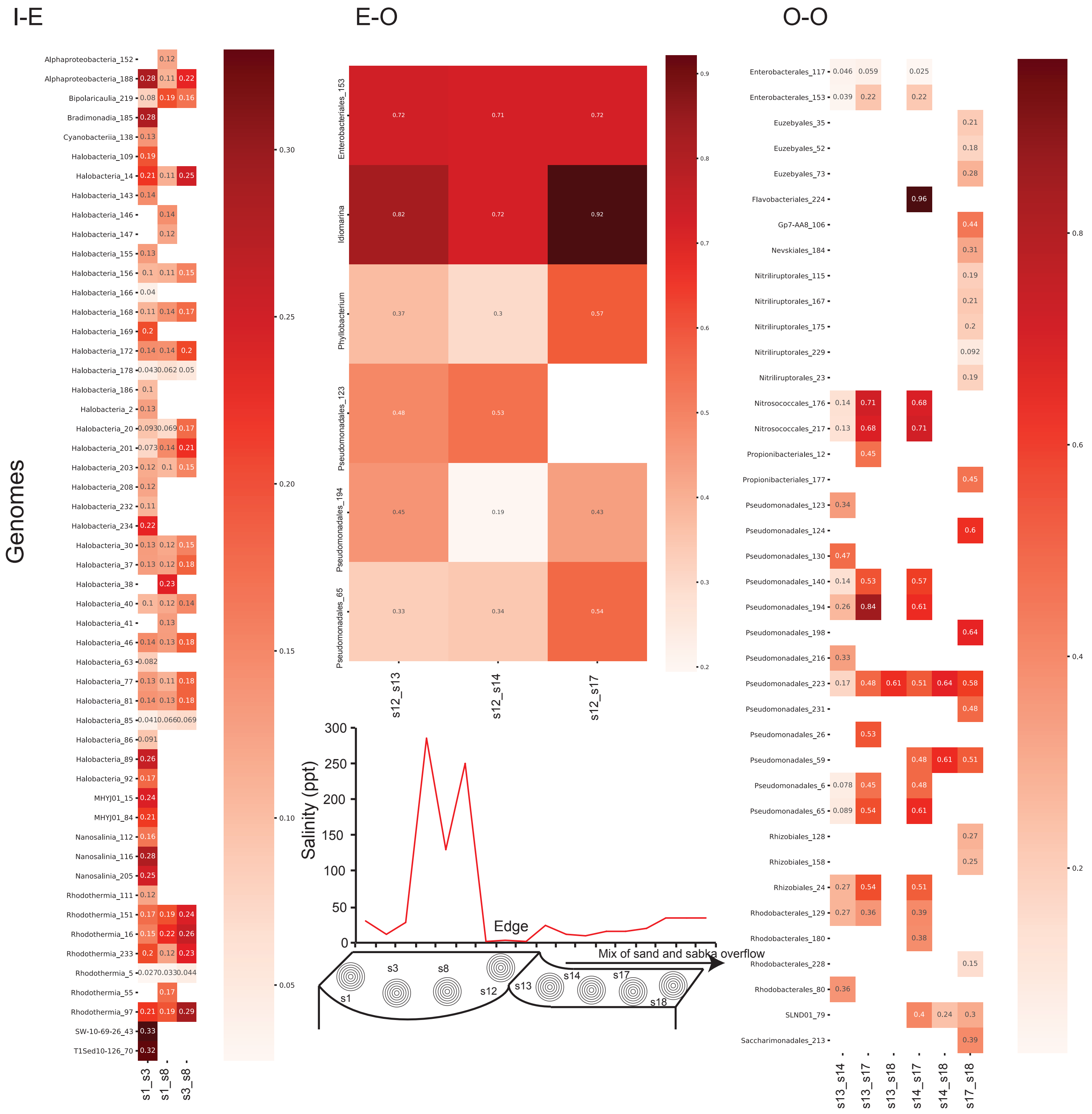

Supplementary Figure 17. Pairwise Fst comparaison along the salinity gradient between sites from inside I-E (s1, s3, s8, s12), outside O-O (s13, s14, s17, s18) and Edge and outside sabkha E-O(s12, s13, s14, s17, s18).

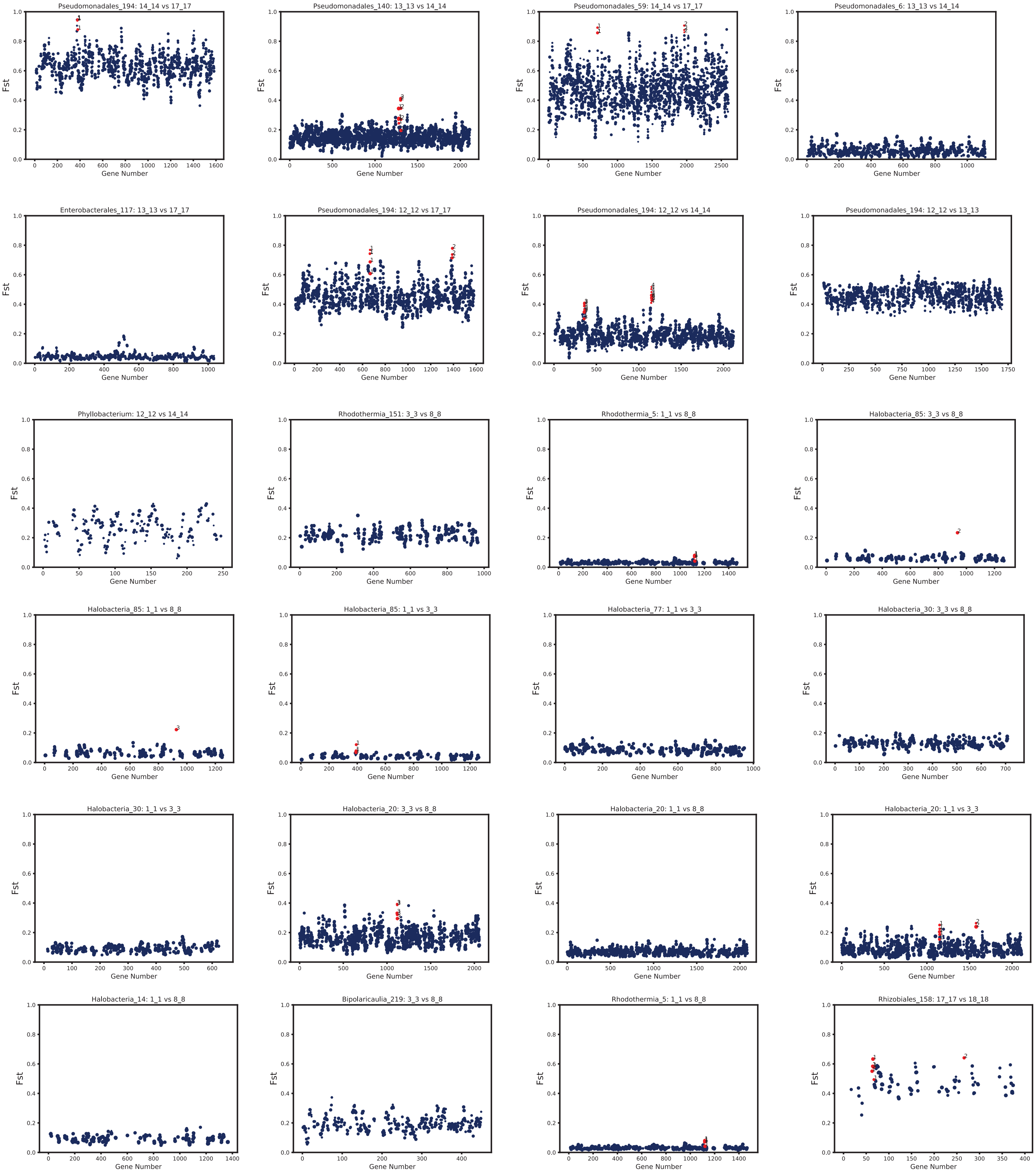

Supplementary Figure 18. Examples of high Fst across populations of sabkha inside, Edge and outside, which is based on a Z-score of 1.5 standard deviations from the mean and a minimum of five consecutive genes before the cut-off decay.

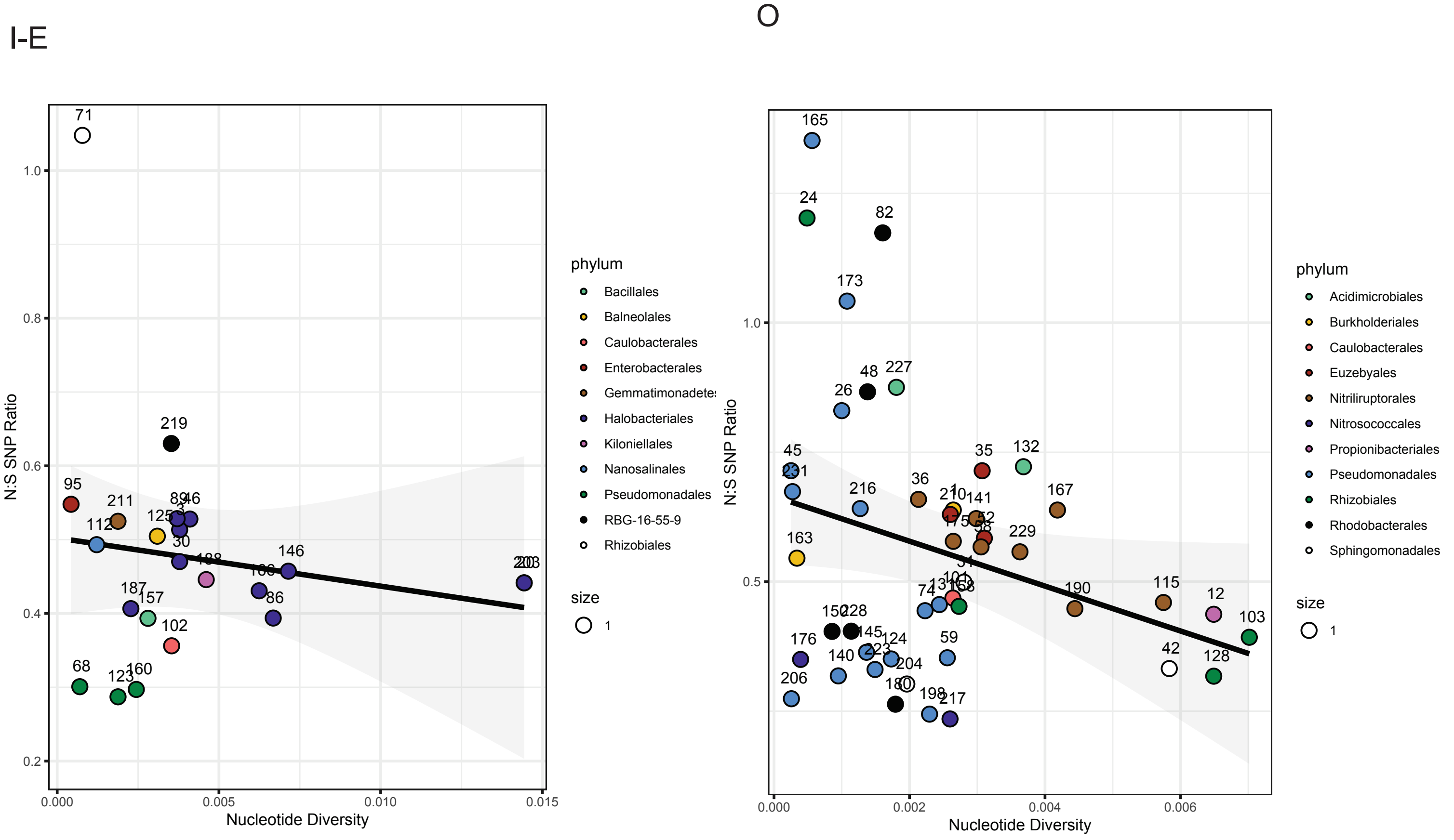

Supplementary Figure 19. Nonsynonymous to synonymous (N: S) SNP ratio correlation decrease with nucleotide diversity for population of sabkha inside (I-E) left panel and Out-side (O) right panel ($p<0.05$)

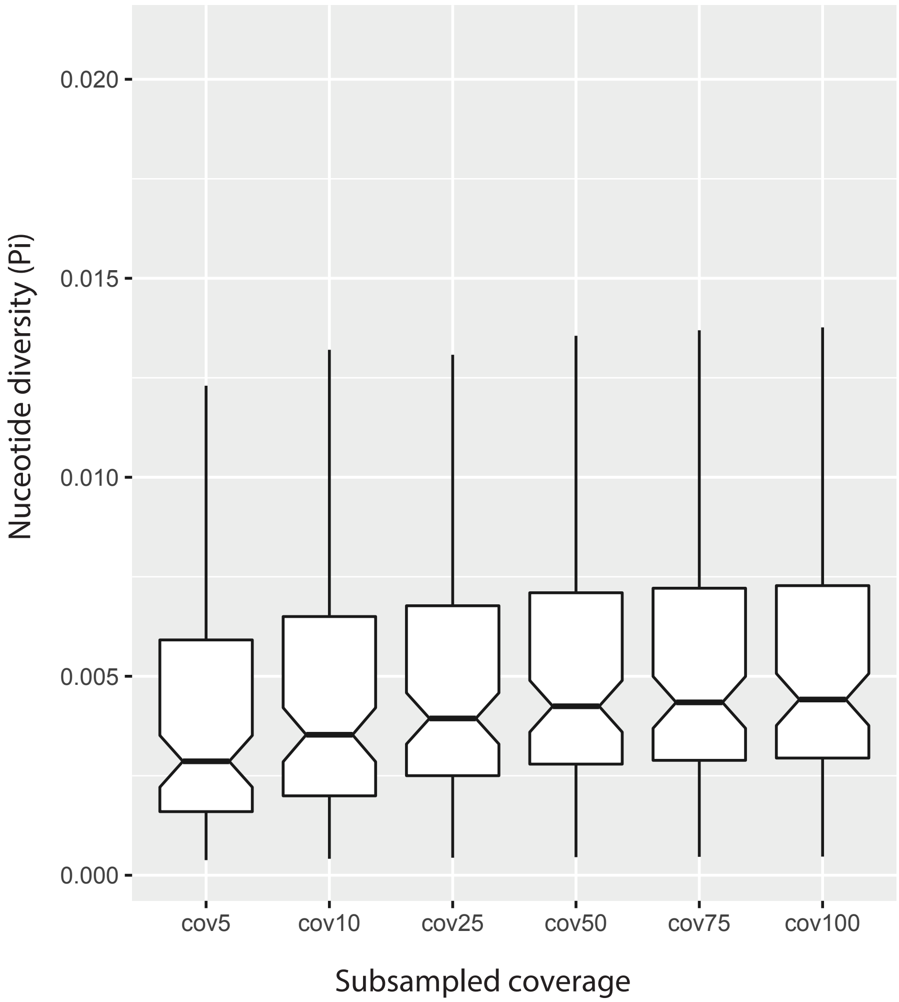

Supplementary Figure 20. Comparing diversity at different subsampling coverage (5,10,25,50,75,100x) across sabkha inside and outside populations.

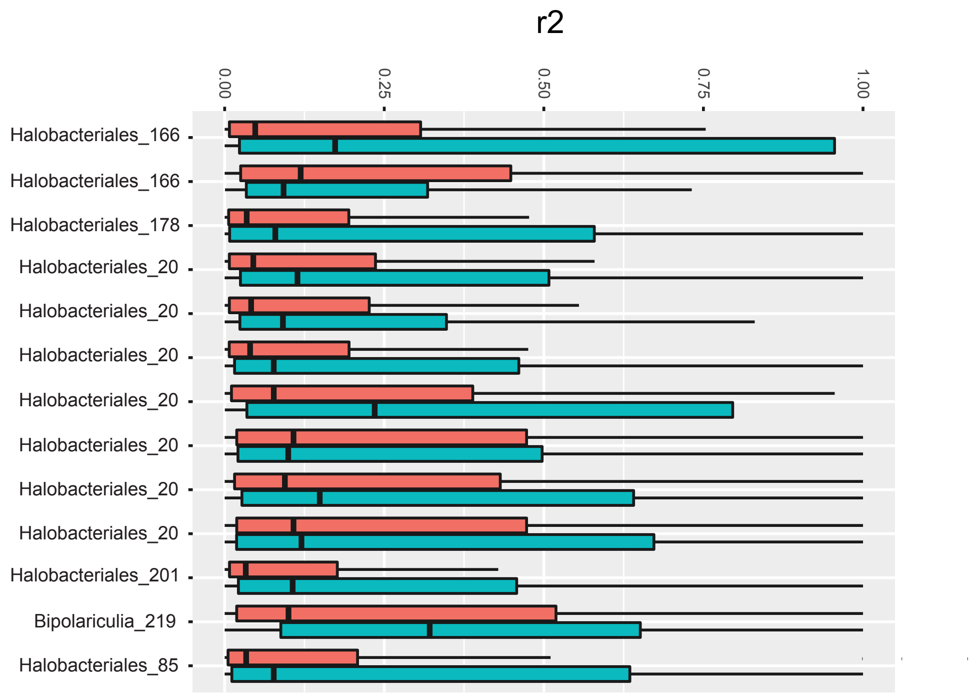

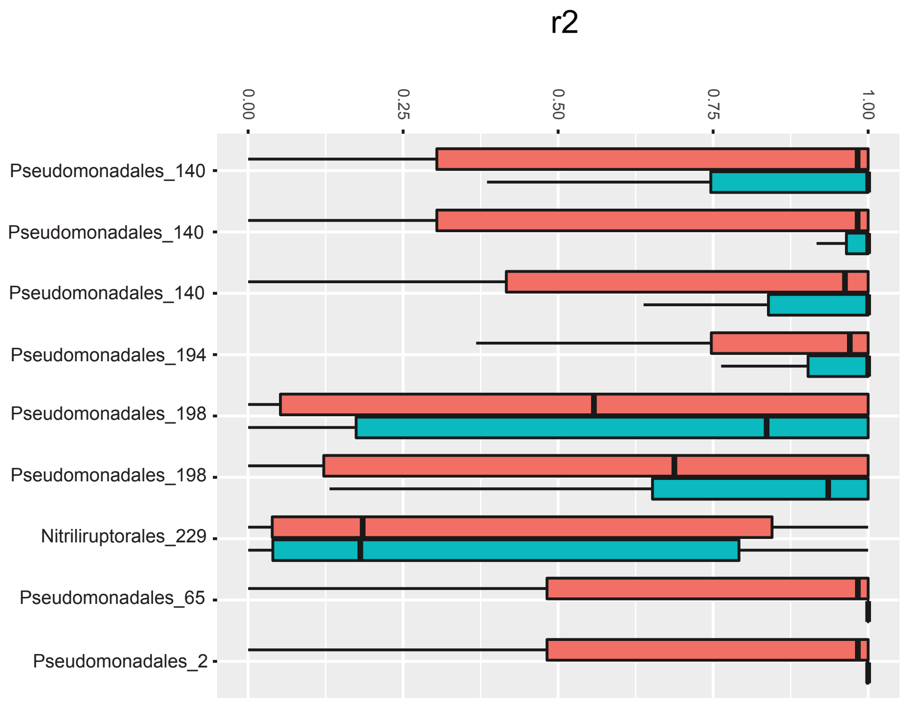

Supplementary Figure 21. Average rate of linkage (r2) of high Fst loci compared to average genome r2 for population of inside (I-E) and outside sabkha (O) ($p<0.05$)

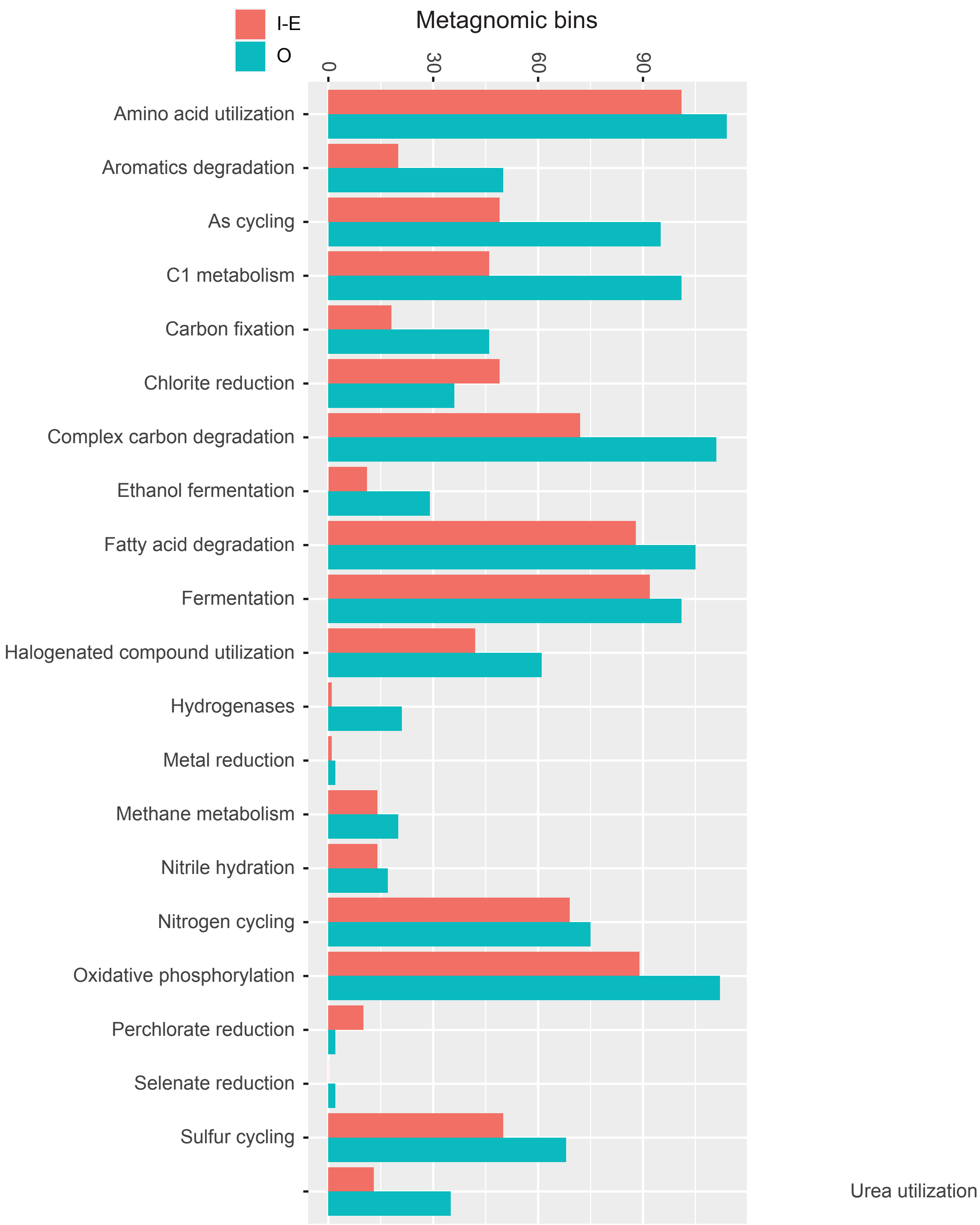

Suplementary Figure 22. Summary of the number of different metagenomic bins, from output of METABOLIC, belonging to different metabolic pathways for inner and outer sabkha.

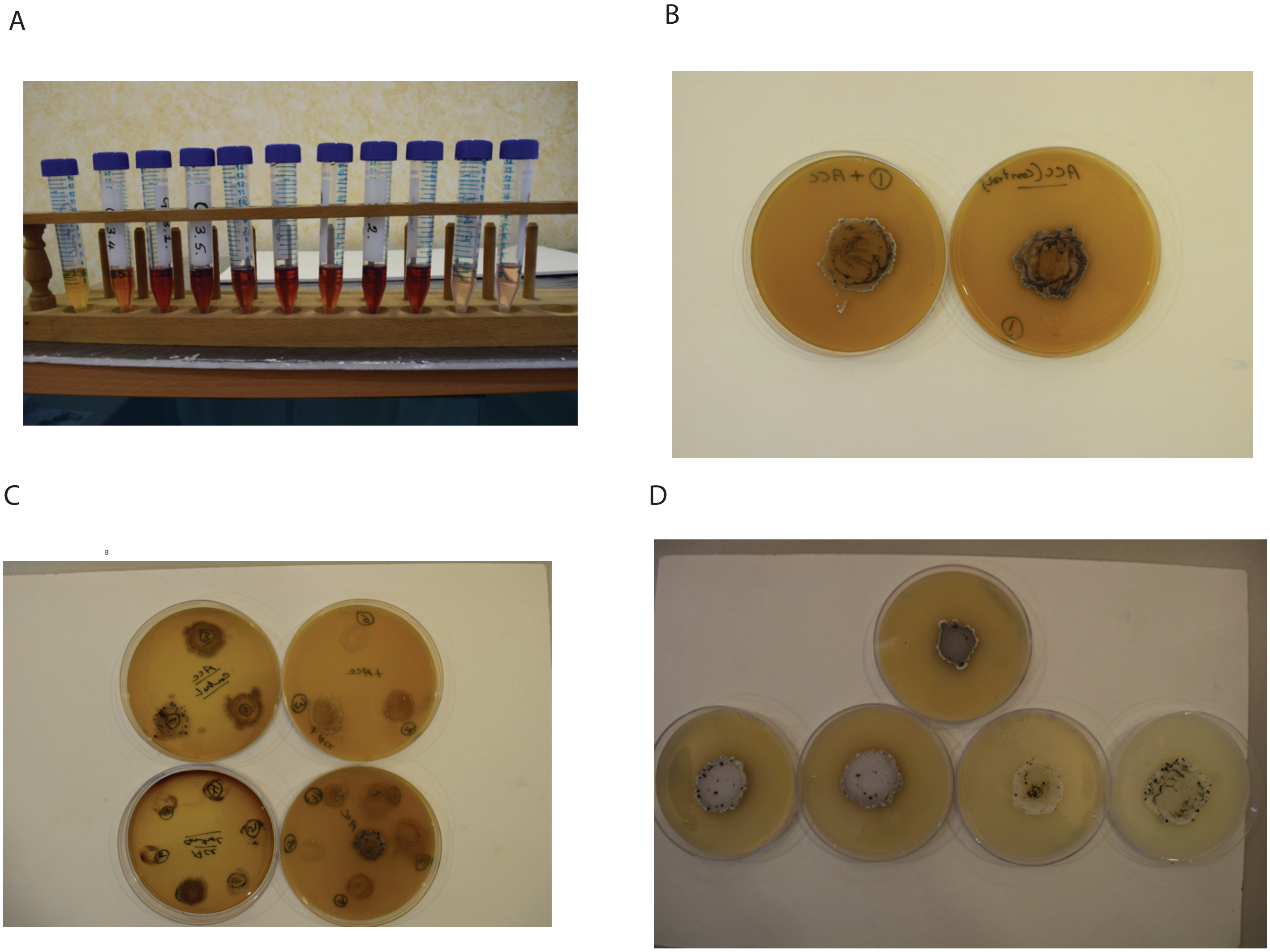

Supplementary Figure 23. Isolation from sabkha isolates of bacteria and actinomycete, with some important properties, which could have potential for agriculture crop improvement. A) The production of IAA bacterial species isolated from Sabkha B) ACC deaminase produced by actinomycete S. mutabilis (left), while the control plate on (Right). C) ACC deaminase produced by different bacteria isolated from Sabka soil, on the right were ACC producers while on the left (Control). D) The growth of Streptomyces mutabilis on SNA medium amended with different concentrations of NaCl: 1.2%, 2.4%, 3.6%, and 4. 8% while the above is SNA without salt (control).

Supplementary methods

Isolation of Bacteria and Actinomycetes from coastal sabkha soil

Soil samples limited to a 3 cm-depth were obtained from costal sabkha in United Arab Emirates (UAE) with clean spade in clean plastic bags. These samples represented three different areas, inner, middle, and outer region of the area. Soil samples were air-dried for 5 days at 28ºC to reduce the number of the contaminant bacteria [1]. Passed through a 5-mm mesh sieve to remove small stones and big soil particles. Fine soil stored in sterile screw-capped jars at 25ºC in the dark, for a week prior to microbiological processing. Bacteria and actinomycete were isolated using the soil dilution plate method [2] on inorganic salt starch agar (ISSA) [3] amended with cycloheximide and nystatin (each 50µg per ml; Sigma – Aldrich) with specific soil pre-treatments [4]. Briefly, the soil pre-treatment involved preparing serial dilutions of the soil suspension by suspending the sample in 6% yeast extract (YE) (Lab M limited) and 0.05% sodium dodecyl sulfate (SDS) (Sigma – Aldrich) for 20 min at 40ºC, and diluting with water to remove other factors promoting bacterial growth or injurious to germinating actinomycete propagules. The YE and SDS were included to increase and decrease the numbers actinomycete and bacteria, respectively [5]. Five replicate plates were used per dilution, which were incubated at 28ºC in dark for seven days. Actinomycete colony was transferred onto oatmeal agar plates supplemented with 0.1% yeast extract (OMYEA) [3]. While the bacterial isolates were transferred onto nutrient agar plates (NA).

We have obtained from the three different areas mentioned before only one actinomycete while eight different bacterial isolates. All were characterized and identified by using 16*S* rRNA gene amplification (Supplementary Table 15). All isolated and identified actinomycete and bacteria were subjected to different biological characters (Supplementary Fig 23).

Production of Indole Acetic Acid (IAA)

Nutrient Agar broth used for bacterial isolates and Starch Nitrate for the actinomycete amended with 5-mmole L – tryptophan was added for each and incubated at 37ºC for bacteria and 28ºC for actinomycete with shaking 180 rpm for 48 h. One ml of culture was centrifuged at 4000 rpm for 10 min and the supernatant was separated. To the supernatant, 2 ml of Salkowiski reagent was added followed by incubation at room temperature under dark conditions. Absorbance of the pink color developed was read at 530 nm. The amount of IAA produced by each isolates was expressed in µg/ml cell protein (Supplementary Fig 23 A) [6]. The production of IAA for bacterial species isolated from Sabkha was measured using optical Density readings at 530 nm for IAA produced by bacterial Isolates and actinomycetes from Sabka (Supplementary Table 15).

Phosphate Solubilization

The isolated bacteria from Sabhka (1 to 9) were spot inoculated onto Pikovaskya's agar medium (Hi – media) amended with 2% (w/v) tricalcium phosphate (TCP) and incubated at 28ºC for 5 days. The development of a clear zone around the colonies was considered as positive phosphate solubilizes (Supplementary Table 15).

In Vitro Assay for Stress Tolerance in Response to Salinity

In order to evaluate salt tolerance characterization, the growth of the endophytic bacteria was observed at 28ºC for 72 h in LB agar medium, supplemented with 2 – 10% Nacl concentration [7]. Moreover, the bacterial suspension of all isolates was used to inoculate sterilized conical flask with nutrient broth medium supplemented with 2 – 10% Nacl, and incubated in a shaker (180 rpm) for 72 h. while for actinomycete Starch Nitrate Agar (SNA) supplemented with the same concentrations of Nacl was used, for control each isolate only nutrient broth medium was used. Then optical density was measured at 600 nm for all sodium chloride concentration compared to the control. (Supplementary Table 15, Supplementary Fig 23 D)

Characterization and Estimation of ACC Deaminase Activity of bacterial isolated from Sabkha soil

The isolated bacteria were screened for ACC deaminase activity on sterile minimal DF (Dworkin and Foster) salts media (DF salts per liter: 4.0 g KH2PO4, 6.0 g Na2HPO4, 0.2 g MgSO4.7H2O, 2.0 g glucose. 2.0 g citric acid and 2.0 gluconic acid with trace elements: 1 mg FeSO4.7H2O, 10 mg H3PO3, 11.19 mg MnSO4.H2O, 124.6 mg ZnSO4.7H2O, 78.22 mg CuSO4.5H2O, 10 mg MoO3, pH 7.2) amended with 3 mM ACC (Sigma – Aldrich), while for control the media was supplemented with (NH4) 2SO4 as a sole nitrogen source instead of ACC [8,9]. The inoculated plates were incubated at 28ºC for 3 days and growth was monitored on a daily basis. Colonies growing on the plates with ACC were considered as ACC deaminase producers (Supplementary Fig 23 B, C)

References

1 Williams, J. Utilisation and taxonomy of the desert grass Panicum turgidum. *Economic Botany* **26**, 13-20 (1972).

2 Johnson, L. F. & Curl, E. A. Methods for research on the ecology of soil-borne plant pathogens. *Methods for research on the Ecology of Soil-Borne Plant Pathogens.* (1972).

3 Küster, E. Outline of a comparative study of criteria used in characterization of the actinomycetes. *International Bulletin of bacteriological nomenclature and taxonomy* **9** (1959).

4 Hayakawa, M. & Nonomura, H. Humic acid-vitamin agar, a new medium for the selective isolation of soil actinomycetes. *Journal of Fermentation Technology* **65**, 501-509 (1987).

5 Nonomura, H. New methods for the selective isolation of soil actinomycetes. *Biology of Actinomycetes' 88, Tokyo* (1988).

6 Pandey, S., Singh, S., Yadav, A. N., Nain, L. & Saxena, A. K. Phylogenetic diversity and characterization of novel and efficient cellulase producing bacterial isolates from various extreme environments. *Bioscience, biotechnology, and biochemistry* **77**, 1474-1480 (2013).

7 Barra, P. J. *et al.* Formulation of bacterial consortia from avocado (Persea americana Mill.) and their effect on growth, biomass and superoxide dismutase activity of wheat seedlings under salt stress. *Applied Soil Ecology* **102**, 80-91 (2016).

8 Dworkin, M. & Foster, J. Experiments with some microorganisms which utilize ethane and hydrogen. *Journal of bacteriology* **75**, 592-603 (1958).

9 Penrose, D. M. & Glick, B. R. Methods for isolating and characterizing ACC deaminase-containing plant growth-promoting rhizobacteria. *Physiologia plantarum* **118**, 10-15 (2003).
